# Hydrogen Tunneling in Catalytic Hydrolysis and Alcoholysis of Silanes

**DOI:** 10.1002/anie.202204558

**Published:** 2022-07-29

**Authors:** Naroa Almenara, Maria A. Garralda, Xabier Lopez, Jon M. Matxain, Zoraida Freixa, Miguel A. Huertos

**Affiliations:** ^1^ University of Basque Country (UPV/EHU) Donostia-San Sebastian 20018 San Sebastián Spain; ^2^ Donostia International Physics Center (DIPC) 20018 San Sebastián Spain; ^3^ IKERBASQUE. Basque Foundation for Science 48013 Bilbao Spain

**Keywords:** Homogeneous Catalysis, Iridium, Quantum Tunneling, Silanes

## Abstract

An unprecedented quantum tunneling effect has been observed in catalytic Si−H bond activations at room temperature. The cationic hydrido‐silyl‐iridium(III) complex, {Ir[SiMe(o‐C_6_H_4_SMe)_2_](H)(PPh_3_)(THF)}[BAr^F^_4_], has proven to be a highly efficient catalyst for the hydrolysis and the alcoholysis of organosilanes. When triethylsilane was used as a substrate, the system revealed the largest kinetic isotopic effect (KIE_Si−H/Si−D_=346±4) ever reported for this type of reaction. This unexpectedly high KIE, measured at room temperature, together with the calculated Arrhenius preexponential factor ratio (A_H_/A_D_=0.0004) and difference in the observed activation energy [(*E* ${}_{a{}_{D}}$ −*E* ${}_{a{}_{H}}$ )=34.07 kJ mol^−1^] are consistent with the participation of quantum tunneling in the catalytic process. DFT calculations have been used to unravel the reaction pathway and identify the rate‐determining step. Aditionally, isotopic effects were considered by different methods, and tunneling effects have been calculated to be crucial in the process.

## Graphical abstract

A “giant” kinetic isotope effect (KIE=346) has been observed for the generation of hydrogen from the hydrolysis of silanes catalyzed by a silyl‐iridium(III) complex. This and other experimental evidence together with theoretical calculations have been used to demonstrate the participation of hydrogen quantum tunneling in this catalytic process.

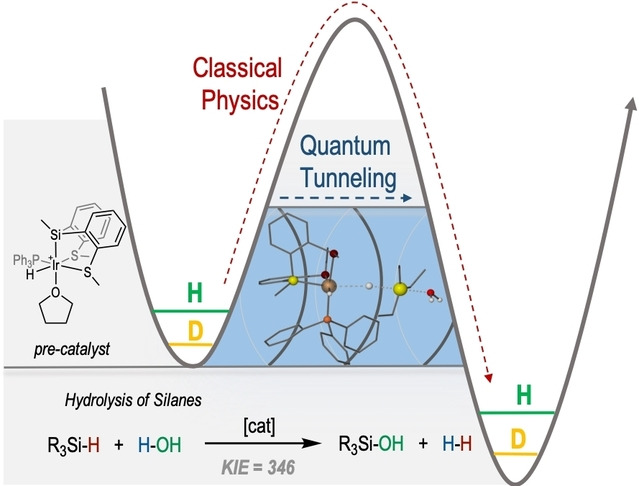

## Introduction

**Figure 1:**
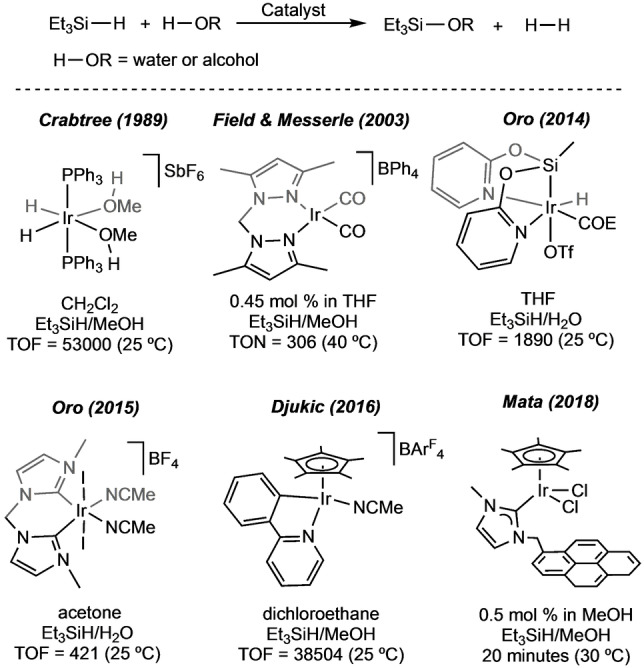
Schematic hydrolysis/alcoholysis of triethylsilane and iridium precatalysts reported for this reaction (TOFs in h^−1^).[^15^, ^16^, ^17^, ^18^, ^19^, ^20^]

The metal‐catalysed hydrolysis and alcoholysis of hydrosilanes are well‐known processes. Early studies focused on the formed silanols/siloxides, considered versatile reaction intermediates in organic transformations.[^1^, ^2^] More recently, the attention drove towards the equimolecular H_2_ evolved through this reaction, which converted hydrosilanes into promising candidates as hydrogen source materials.^[3]^ The hydrolysis of the Si−H bonds is a thermodynamically favourable process, but it suffers from slow kinetics.^[4]^ For this reason, many catalytic systems based on transition metals such as gold,[^5^, ^6^] iron,^[7]^ ruthenium,[^8^, ^9^] rhenium,[^10^, ^11^] rhodium[^12^, ^13^] and iridium[^14^, ^15^, ^16^, ^17^, ^18^, ^19^, ^20^] have been developed. Among them, the iridium‐based catalysts stand out due to their high activities (Figure 1). In 1989, Luo and Crabtree reported on the high catalytic activity of a cationic dihydride‐Ir^III^ complex in the methanolysis of triethylsilane (Figure 1).^[15]^ In 2014, Oro et al. reported on a neutral hydrido‐silyl‐Ir^III^ complex, which behaves as an excellent catalyst in the hydrolysis of Et_3_SiH (Figure 1).^[17]^ Among the different Ir^III^ catalytic systems reported to date, cationic complexes seem to outperform neutral derivatives in both hydrolysis and alcoholysis of silanes (Figure 1).[^15^, ^19^] The efficiency of the cationic iridium systems is attributed to the electrophilic character of the metal centre.[^15^, ^19^] With these systems the hydrosilane activation should occur by coordination to the electrophilic metal centre, as σ‐H‐SiR_3_ ligand, rather than through oxidative addition.

**Scheme 1:**
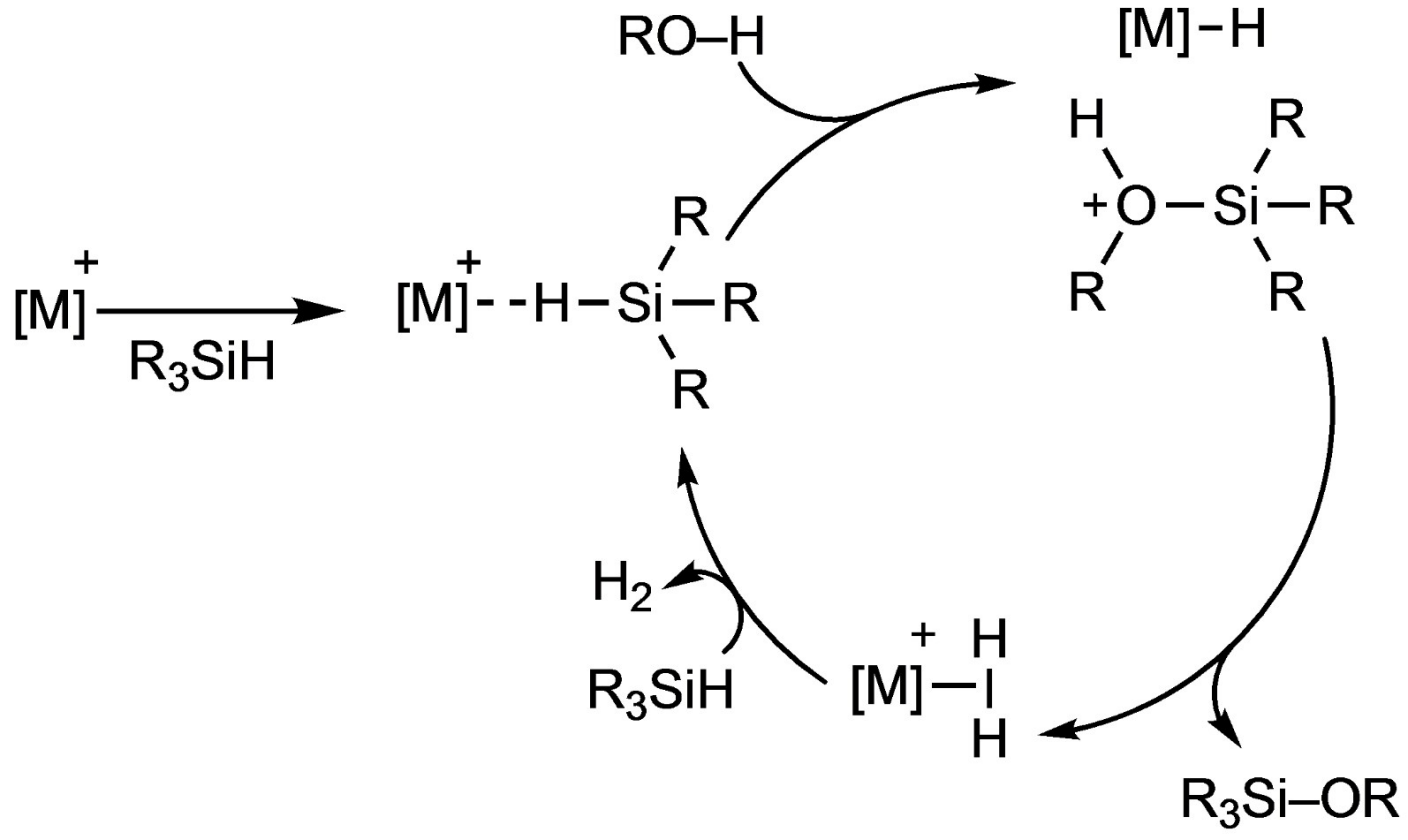
Proposed electrophilic mechanism for the hydrolysis/alcoholysis of silanes.

The catalytic cycle has been described as an outer‐sphere nucleophilic attack of alcohol or water on the activated silicon center, generating an iridium‐hydride and a solvent‐stabilized silylium cation, which immediately reacts to form an iridium‐dihydrogen complex and the corresponding silanol or silylether. Liberation of hydrogen from the metal center and coordination of a new hydrosilane close the catalytic cycle, requiring only one vacant coordination site on the metal to proceed (Scheme 1).

This mechanism, initially proposed by Luo and Crabtree in 1989,^[15]^ has been accepted for the hydrolysis and alcoholysis of hydrosilanes involving different electrophilic catalysts.[^7^, ^17^, ^18^, ^19^, ^20^, ^21^, ^22^, ^23^, ^24^, ^25^, ^26^] While there are examples of electrophilic mechanism for the hydrolysis/alcoholysis of silanes with other transition metals,[^7^, ^21^, ^22^, ^23^, ^24^, ^25^, ^26^] mechanistic studies involving cationic iridium complexes are still very attractive due to its high capability to transfer electrophilicity inducing Si−H activation.[^27^, ^28^] Considering iridium(III) complexes, this kind of mechanism has been well studied theoretically by Oro et al. in 2014^[17]^ and Mata et al. in 2018.^[20]^ Moreover, in 2016, the group of Djukic has extensively studied the mechanism of the alcoholysis of triethylsilane experimentally. The work reported by Djukic et al. includes the full characterization of a cationic iridium η^1^‐silane complex and other possible intermediates of this reaction, as for example hydride species.^[19]^

In the last years, our research group has been working on the synthesis and applications of rhodium and iridium complexes with silyl‐thioether multidentate ligands. This kind of ligands proved useful to stabilize unsaturated rhodium and iridium cationic complexes leaving a coordinative vacant site in trans with respect to the coordinated silicon center (often occupied by a solvent molecule).[^29^, ^30^] Assuming an electrophilic mechanism for the hydrolysis/alcoholysis of silanes (Scheme 1), unsaturated iridium(III) compounds are perfectly suited to activate hydrosilane through coordination prompting the subsequent nucleophilic attack of water/alcohol.

**Figure 2:**
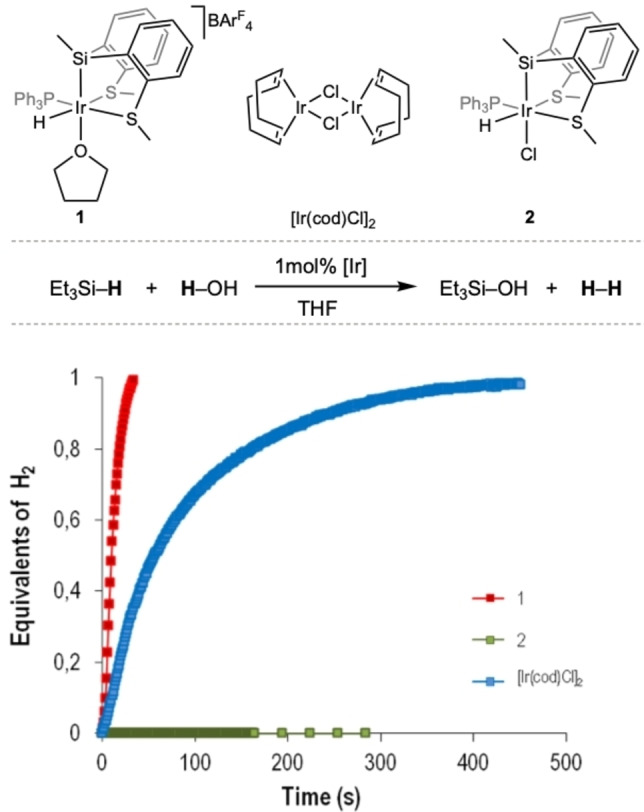
Iridium precatalysts used in this work, hydrolysis of Et_3_SiH catalyzed by **1**, **2** and [Ir(cod)Cl]_2_ and reaction profiles (equiv. of H_2_ generated vs time). Reaction conditions: Et_3_SiH (0.25 mmol), H_2_O (2.5 mmol), catalyst (0.0025 mmol based on iridium) in THF (1 mL) at 25 °C. H_2_ production calculated by continuous monitoring of the pressure evolution using a pressure transducer (Man on the Moon X102 kit).

Herein we present combined experimental and theoretical studies on the catalytic activity of a cationic iridium species {Ir[SiMe(o‐C_6_H_4_SMe)_2_](H)(PPh_3_)(THF)}[BAr^F^_4_] (**1** in Figure 2) in the hydrolysis and alcoholysis of silanes. The involvement of hydrogen tunneling in the rate‐determining step (RDS) of the process permitted us to explain the high catalytic activity and the unexpectedly large KIE${}_{\mathrm{Et}{}_{3}\mathrm{SiH}/\mathrm{Et}{}_{3}\mathrm{SiD}}$ observed at room temperature.[^31^, ^32^, ^33^, ^34^]

## Results and Discussion

Iridium(III) compounds **1** and **2** ^[30]^ have been studied as precatalysts for the hydrolysis of Et_3_SiH in THF under standard reaction conditions (1 mol % catalyst, [Et_3_SiH]=0.25 M, 10 equiv H_2_O). The parent iridium dimer [Ir(cod)Cl]_2_ has also been included in the initial screening for comparative purposes.^[14]^ The reaction profiles obtained (equiv. of H_2_ vs time) are shown in Figure 2. The hydrogen evolution during the reaction was continuously monitored through a pressure transduced implemented in the Man on the Moon X102 kit (see Supporting Information for details).

**Figure 3:**
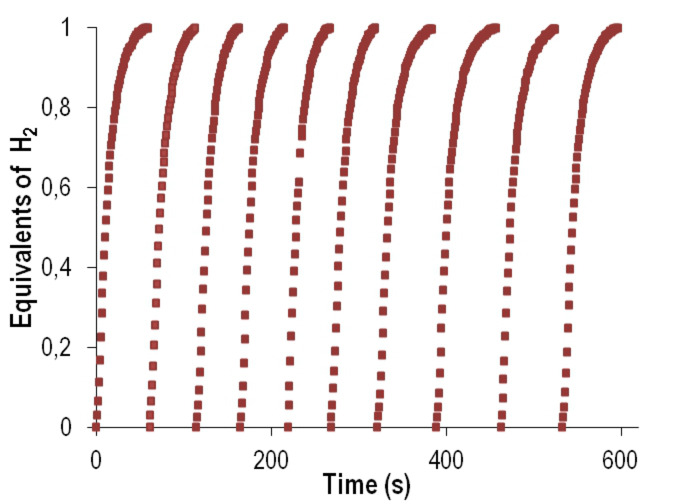
Reaction profiles obtained by successive additions of 0.25 mmol of Et_3_SiH to a THF solution containing 0.0025 mmol of compound **1** and 5.5 mmol of H_2_O. H_2_ production calculated by continuous monitoring of the pressure evolution using a pressure transducer (Man on the Moon X102 kit).

The results obtained showed that no hydrogen was liberated when the saturated complex **2** was used as precatalyst. This result indicates that a vacant coordination site on the catalyst is required for the reaction to proceed. In contrast, compound **1**, containing a labile solvent molecule, was a very effective catalyst for the hydrolysis of Et_3_SiH, liberating 1 equiv of H_2_ in only 35 seconds (Table 1, entry 1). The activity observed (turnover frequency calculated at 50 % of conversion, TOF_1/2_) was 2.8 times larger than that observed for the dimeric iridium precursor [Ir(cod)Cl]_2_ under identical reaction conditions. ^1^H and ^1^H‐^29^Si HMBC NMR analysis of evaporated samples at the end of the reaction permitted us to identify triethylsilanol as the only silane‐containing product (Figure S1, Supporting Information). The robustness of the catalytic system derived from **1** was confirmed through successive additions of Et_3_SiH. The sequential reaction profiles obtained showed that the catalyst maintained its activity for at least 10 successive cycles (Figure 3).

**Table 1:** Catalytic hydrolysis/alcoholysis of hydrosilanes.^[a]^

Entry	Catalyst	Silane	Nucleophile	TOF_1/2_ (h^−1^)^[b]^
1	**1**	Et_3_SiH	H_2_O	20 385(2850)
2	**2**	Et_3_SiH	H_2_O	0
3	**[Ir(cod)Cl]_2_**	Et_3_SiH	H_2_O	6276^[c]^
4	**1**	Me_2_PhSiH	H_2_O	13 5678(20 509)
5	**1**	MePh_2_SiH	H_2_O	44 776(297)
6	**1**	Ph_3_SiH	H_2_O	950(29)
7	**1**	Et_3_SiH	MeOH	13 107(671)
8	**1**	Et_3_SiH	EtOH	11 952(289)
9	**1**	Et_3_SiH	iPrOH	1301(47)

[a] Reaction conditions: Silane (0.25 mmol), H_2_O or alcohol (2.5 mmol), 1 mol % of catalyst (based on iridium) in 1 mL of THF at 25 °C. [b] TOF_1/2_: turnover frequency calculated at a reaction time corresponding to 50 % of conversion, values averaged from 3 runs, errors in parenthesis. [c] Only one run.

The hydrolytic reaction with precatalyst **1** was also studied using a range of hydrosilanes under standard conditions (see Table 1). In all cases, the corresponding silanol was obtained as the sole reaction product, as confirmed by ^1^H and ^1^H‐^29^Si HMBC NMR analysis of evaporated reaction mixtures (see Figures S2–S4, Supporting Information). The results obtained showed a clear electronic influence of the silane substituents on the reaction rate (entries 4, 5 and 6, Table 1), being faster when increasing the electron‐donating nature of the substituents except for Et_3_SiH. The low activity of Et_3_SiH compared with Me_2_PhSiH and MePh_2_SiH could be attributed to the larger buried volume of the latter which would hinder it from approaching the metal center (see Figure S8, Supporting Information).^[35]^

The catalytic activity of compound **1** towards the alcoholysis of Et_3_SiH was also explored (Table 1, entries 7–9, and Figure S10, Supporting Information). The results obtained showed that in all cases one equivalent of hydrogen was liberated, and the corresponding silylethers were the only silicon‐containing reaction products, as confirmed by ^1^H and ^1^H‐^29^Si HMBC NMR analysis of evaporated reaction mixtures (Figures S5–S7, Supporting Information). The reaction proceeded at similar rates for the primary alcohols assayed (entries 7 and 8, Table 1). It is worth noticing that, even if at a much‐reduced rate, compound **1** was also an effective catalyst when a secondary alcohol (iPrOH) was used as a nucleophile.

These results positioned the cationic iridium complex **1** among the most active catalytic systems for the hydrolysis and alcoholysis of organosilanes.[^5^, ^6^, ^7^, ^8^, ^9^, ^10^, ^11^, ^12^, ^13^, ^14^, ^15^, ^16^, ^17^, ^18^, ^19^, ^20^]

Induction periods were observed in most of the catalytic reactions. The addition of excess Hg during the hydrolysis of triethylsilane led to similar results, and no darkening of the solution was observed, suggesting the homogeneous nature of the catalytic process.^[36]^ Therefore, the induction period was, at this point, tentatively attributed to decoordination of THF being required to obtain an active species to start the catalytic reaction.

**Figure 4:**
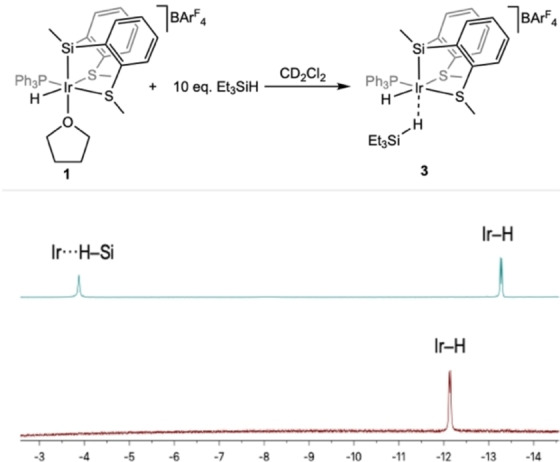
Scheme of the reaction of **1** with Et_3_SiH in CD_2_Cl_2_ and hydride region of the ^1^H NMR spectra of **1** (bottom) and after addition of 10 equivalents of Et_3_SiH (top), at −90 °C.

A series of NMR experiments were performed to obtain some information about the reaction mechanism. Initially, the reactivity of **1** toward H_2_O was studied. **1** was reacted with 100 equivalents of H_2_O in THF‐d_8_ for 12 hours. ^1^H NMR spectrum of the starting compound **1** remained apparently unaltered apart from a small displacement (0.03 ppm) and broadening of the signal attributed to the coordinated hydride (Figure S20, Supporting Information). This can be explained by Ir−H/H_2_O exchange via short‐lived dihydrogen complexes^[37]^ or because an Ir−THF⇌Ir−OH_2_ equilibrium is taking place. Both possibilities would involve a H_2_O coordination to the metal center. When the same reaction was performed in CD_2_Cl_2_, displacements of the Ir−H signal (0.82 ppm) and Si‐CH_3_ signal (0.1 ppm) were observed (Figure S21). This fact reinforces the idea of an Ir−THF⇌Ir−OH_2_ equilibrium. No hydrogen evolution was observed during these experiments, therefore, a mechanism operating through Ir^III^‐OH intermediates, as proposed by Luo et al.,^[38]^ can be excluded. To study the ability of complex **1** to activate Et_3_Si−H, as proposed within the electrophilic mechanism, compound **1** was reacted with 10 equivalents of Et_3_Si−H in THF‐d_8_. ^1^H NMR spectrum shows a 1.1 ppm displacement on the Ir−H signal, which could indicate the formation of a cationic iridium η^1^‐silane complex. Unfortunately, sigma interaction between Et_3_SiH and **1** was not observed in a static ^1^H NMR (−90 °C) in this solvent. Triethylsilyl iridium complex is not observed, which would exclude a mechanism via oxidative addition/reductive elimination steps. The same reaction (**1** and 10 equivalents of Et_3_Si−H) was studied in CD_2_Cl_2_ at −90 °C. The hydride region of the ^1^H NMR spectrum showed the transformation of the signal attributed to the hydride of **1** (*δ*=−12.13, *J*_H‐P_=18.5 Hz), into two new signals at −3.88 and −13.27 ppm, respectively (Figure 4 and Figures S22 and S23 in Supporting Information). The former signal was a singlet accompanied by the characteristic ^29^Si satellites (*J*_H‐Si_=98 Hz) (Figure S23, Supporting Information). It was assigned to an Ir−H−Si hydride, by comparison with a cationic iridium η^1^‐silane complex previously reported by Brookhart.^[39]^ The resonance at higher field resolved into a doublet (*J*_H‐P_=16.3 Hz), and resembled that of the parent compound **1**. Therefore, it was assigned to a terminal hydride trans to a sulfur atom and *cis* to PPh_3_. Additionally, the ^31^P{^1^H} NMR spectrum showed the transformation of the original singlet at 19.7 ppm into a new singlet at 16.0 ppm (Figure S24, Supporting Information).

These observations are consistent with the formation of compound **3** (Figure 4) containing a η^1^‐coordinated hydrosilane,[^19^, ^39^] which would demonstrate the capacity of **1** to promote the electrophilic activation of hydrogen‐silicon bonds as proposed within the electrophilic mechanism.

In an effort to obtain evidence of the origin of the formed hydrogen, several catalytic reactions using Et_3_SiH/H_2_O, Et_3_SiH/D_2_O, and Et_3_SiD/H_2_O (ratio silane/water of 1/10 in all cases) were performed in a sealed NMR tube. As expected, using Et_3_SiH/H_2_O, only H_2_ was observed in the ^1^H NMR spectrum (Figure S19, Supporting Information). Therefore, the relative integration of the H_2_ signals of the spectra compared to that of triethoxysilanol (used as internal reference) were used to calculate the total amount of gas dissolved in a fully converted sample. Under identical experimental conditions, the hydrolysis of Et_3_SiD with H_2_O led to the formation of H_2_ mainly (Figure S19, Supporting Information). Finally, when Et_3_SiH/D_2_O are used as reactants, a H_2_/HD mixture in a ratio of 14/86 was observed by ^1^H NMR (Figure S19, Supporting Information). According to our calculations, this mixture corresponds to approximately 10 % of the total gas evolved, being the main component ^1^H NMR‐blind D_2_ (amount of D_2_ estimated of 90 %, see Supporting Information for more details). The formation of H_2_ (≈1.4 %) would be due to traces of H_2_O. These results indicate that, eventually, the isotope composition of the hydrogen released is determined by the water isotope used, which may be due to quick exchange between Ir−H(D) and the water protons as previously reported by Himeda et al. for iridium‐catalyzed formic acid dehydrogenation reactions.^[40]^

**Figure 5:**
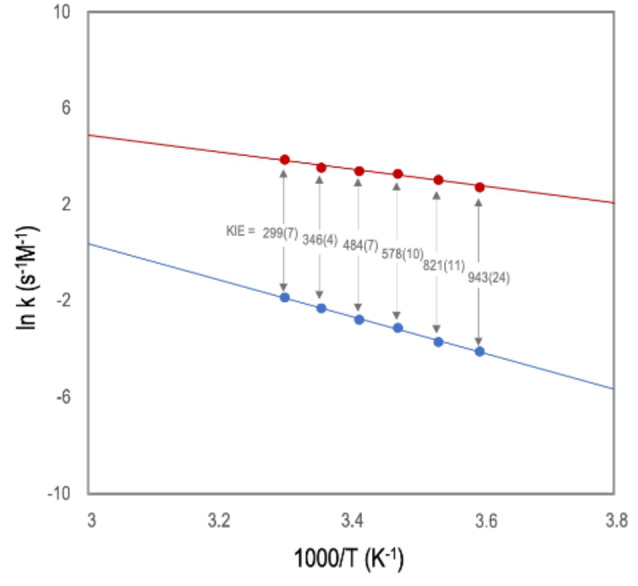
Arrhenius plots for the hydrolysis of Et_3_Si−H (red) and Et_3_Si−D (blue) catalyzed by **1**, leading to values of A (from the intercept) and *E*_a_ (from the slope). From the differences of the plots the KIE at each temperature is calculated, errors in parenthesis.

The results obtained using **1** as precatalyst showed a first‐order dependence of the reaction rate on Et_3_SiH and catalyst concentration, but a zero‐order dependence on H_2_O (*v*=*k* [**1**] [Et_3_SiH]) (Figures S11–S12, Supporting Information). Kinetic isotopic effect (KIE) was analyzed, using compound **1**, on both the hydrolysis and the methanolysis of Et_3_SiH. The results obtained showed no effect on the reaction rate when a deuterated nucleophile was used (KIE${}_{H{}_{2}O/D{}_{2}O}$ =KIE_MeOH/MeOD_=1), which is consistent with the zero‐order dependence of the reaction rate on H_2_O concentration (see above). Surprisingly, extraordinarily high KIEs (25 °C) were calculated when the reaction rate using Et_3_SiD was compared to that of Et_3_SiH (KIE${}_{\mathrm{Et}{}_{3}\mathrm{SiH}/\mathrm{Et}{}_{3}\mathrm{SiD}}$ (25 °C)=346±4 (hydrolysis); KIE${}_{\mathrm{Et}{}_{3}\mathrm{SiH}/\mathrm{Et}{}_{3}\mathrm{SiD}}$ (25 °C)=143±1 (methanolysis), see Figures S14 and S16, Supporting Information. These results clearly point to H−Si bond activation being involved in the rate‐determining step (RDS) of the catalytic reaction, and suggest the possible participation of hydrogen quantum tunneling in this step of the process.[^31^, ^32^, ^33^, ^34^] Rate constants and KIEs were also calculated for the catalytic hydrolysis of other hydrosilanes (KIE${}_{\mathrm{PhMe}{}_{2}\mathrm{SiH}/\mathrm{PhMe}{}_{2}\mathrm{SiD}}$ =4.3±0.5; KIE${}_{\mathrm{Ph}{}_{3}\mathrm{SiH}/\mathrm{Ph}{}_{3}\mathrm{SiD}}$ =16.8±0.2 (Figure S17, Supporting Information). These values show that the KIE (and probability of quantum tunneling) strongly depends on the hydrosilane. Considering that with deuterated derivatives the probability of hydrogen tunneling is very low, the rate constants (*k* ${}_{\mathrm{PhMe}{}_{2}\mathrm{SiD}}$ =50.6±1.5 s^−1^ M^−1^; *k* ${}_{\mathrm{Et}{}_{3}\mathrm{SiD}}$ =0.10±0.01 s^−1^ M^−1^) would be indicative of a much lower classic barrier for the former. Therefore, the relatively low KIE observed for PhMe_2_SiH(D) could be explained by an increase of the classical component to the overall rate that outruns tunneling. These observations would be in agreement with our mechanistic proposal (see below) revealing the importance of the Ir⋅⋅⋅H⋅⋅⋅Si distances in the active species for the reaction to proceed through quantum tunneling. When [Ir(cod)Cl]_2_ was used as precatalyst, KIE${}_{H{}_{2}O/D{}_{2}O}$ =1.10±0.03 and KIE${}_{\mathrm{Et}{}_{3}\mathrm{SiH}/\mathrm{Et}{}_{3}\mathrm{SiD}}$ =2.26±0.06 (Figure S18, Supporting Information) were measured. These results also pointed to a related mechanism, with H−Si bond activation being involved in the RDS, but hydrogen quantum tunneling could not be claimed in this case. In fact, a comparison of rate constants between precatalysts **1** and [Ir(cod)Cl]_2_ using Et_3_SiD as a nucleophile (*k*(**1**)=0.101 s^−1^ M^−1^; *k*([Ir(cod)Cl]_2_)=8.45 s^−1^ M^−1^) reinforces the idea that the high activities observed for our system are caused by a quantum tunneling mechanism, strongly inhibited when deuterosilanes were used as nucleophiles. No tunneling has been observed for the methanolysis of Et_3_SiH(D) using the cationic iridium(III) complex [IrCp*(phenylpiridine)(MeCN)]^+^.^[19]^ To reinforce the hypothesis that quantum tunneling participates in the RDS of this reaction, we looked for additional experimental pieces of evidence. As generally accepted, the observation of an unusually low Arrhenius preexponential factor ratio (A_H_/A_D_<0.7) and a difference in the observed activation energy larger than the ones expected from zero‐point energy (*E* ${}_{a{}_{D}}$ −*E* ${}_{a{}_{H}}$ >5.02 kJ mol^−1^) would support this idea.[^41^, ^42^] Therefore, the hydrolysis of Et_3_SiH and Et_3_SiD were studied at different temperatures (5–30 °C) (Figures S13 and S15, Supporting Information). Comparative Eyring and Arrhenius analyses of the data obtained showed linear but non‐parallel correlations (Figure 5). From these data a A_H_/A_D_ ratio of 0.0004±0.0003 and *E* ${}_{a{}_{D}}$ −*E* ${}_{a{}_{H}}$ =34.1±0.2 kJ mol^−1^ were calculated. These results are consistent with an important contribution of hydrogen tunneling to the process and most probably being responsible for the extraordinarily high activities observed when non‐deuterated nucleophiles were used. Moreover, using the Eyring analysis, the activation barriers were determined: Et_3_SiH (Δ*H* ^≠^=27±2 KJ mol^−1^, Δ*S* ^≠^=−125±7 J mol^−1^ K^−1^ and Δ*G*(298)^≠^=64±4 KJ mol^−1^); Et_3_SiD (Δ*H* ^≠^=60±2 KJ mol^−1^, Δ*S* ^≠^=−60±6 J mol^−1^ K^−1^ and Δ*G*(298)^≠^=79±4 KJ mol^−1^). The significant negative activation entropy suggests a transition state considerably more ordered than the reactants, which agrees with a S_N_2‐type mechanism in the RDS, as has been previously reported for an iron catalyst.^[7]^ Moreover, negative entropies are known to be an indication for tunneling.^[43]^

**Figure 6:**
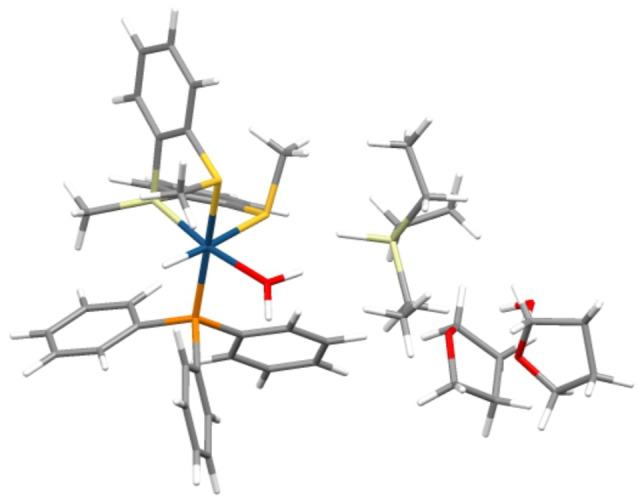
Calculated structure of the **C_0_** reactant complex.

The high activities observed in the hydrolysis and alcoholysis of hydrosilanes, together with the experimental pieces of evidence pointing to the involvement of hydrogen tunneling in the process, prompted us to study the reaction from a theoretical point of view. DFT calculations[^44^, ^45^] have been carried out using the M06^[46]^ functional and considering the implicit solvent effect by means of IEFPCM^[47]^ method for both geometry optimizations and frequency calculations, as implemented in Gaussian 16 program package.^[48]^ According to our calculations, described in the Supporting Information, the reactant complex would be the **C_0_** complex (Figure 6).

**Figure 7:**
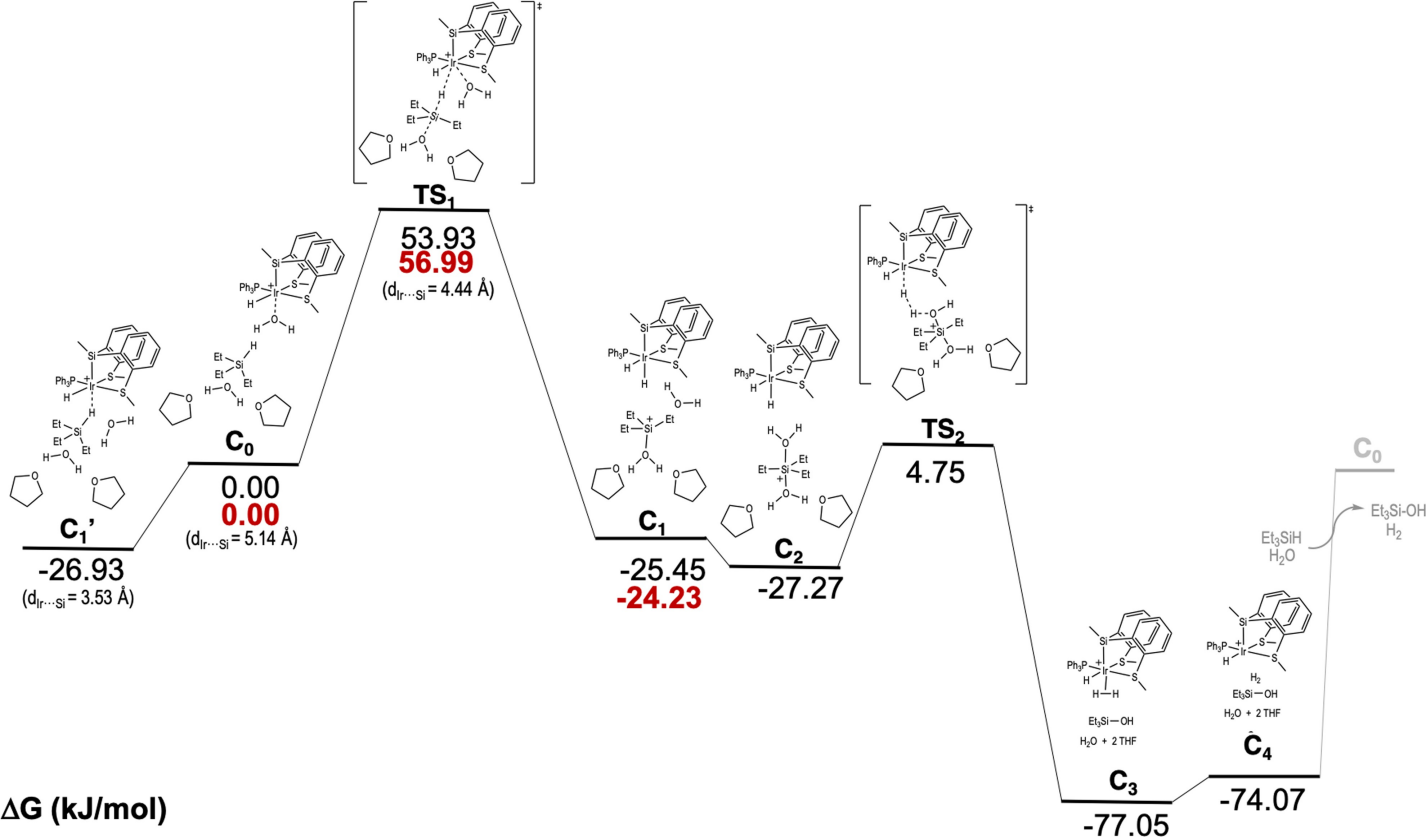
Full Gibbs Free Energy Surface for the studied reaction mechanism, in kJ mol^−1^. Red values correspond to deuterated species.

This reactant complex is an iridium‐aquo complex, formed by ligand exchange from the starting iridium‐THF compound. A triethylsilane molecule (not located in the first coordination sphere; *d* Ir⋅⋅⋅Si=5.14 Å), which contains a back‐side H_2_O molecule solvated with two THF solvent molecules, is also part of **C_0_**. Starting from this reactant complex, the reaction may take place, and the calculated full reaction mechanism is depicted in Figure 7.^[49]^ The optimized geometry for **C_0_**, showed that the hydridic hydrogen atom of the hydrosilane is oriented towards one of the protic hydrogens of the coordinated H_2_O (*d* H⋅⋅⋅H=1.80 Å), which may indicate a kind of H^∂+^−H^∂−^ interaction. Indeed, calculated Natural Bond Orbital charges (NBO charges)[^50^, ^51^, ^52^] show values of 0.541 e and −0.271 e, for the H−OH and Si−H, respectively. These values show that there is in fact a stabilizing H^∂+^−H^∂−^ interaction that attracts the triethylsilane moiety towards the catalyst. In order to check for the importance of this interaction along the reaction mechanism, we have calculated the partial charges and interatomic distances all along the reaction steps, and the results are collected in Table 2.

**Table 2:** Calculated Natural Bond Orbital charges (NBO)[^49^, ^50^, ^51^] for the protic and hydridic hydrogens, and the interatomic distances between them.

	H^∂+^ (e)	H^∂−^ (e)	d (H^∂+^–H^∂−^) [Å]
**C_1′_**	0.518	−0.290	4.49
**C_0_**	0.541	−0.271	1.80
**TS_1_**	0.511	−0.372	1.68
**C_1_**	0.518	−0.124	1.71
**C_2_**	0.547	−0.118	1.57
**TS_2_**	0.379	−0.139	0.99
**C_3_**	0.147	0.115	0.83
**C_4_**	0.001	−0.001	0.74

One of the possible paths starting from **C_0_** is the complete substitution of the coordinated H_2_O molecule by the Et_3_SiH, leading to the formation of **C_1_′** complex. In this vein, the H^∂+^−H^∂−^ interaction found in **C_0_** is broken, being the interatomic distance too long for such interaction (4.49 Å). This structure can be described as an iridium η^1^‐silane complex where the Si⋅⋅⋅Ir distance is 3.53 Å, which proved to be a stable structure. The consequence of such stability is that all attempts to calculate a nucleophilic attack to the Si atom eventually ended on the same structure, which shows that this is a non‐reactive path for these species. It is worth noting that analogue species have been commonly proposed to be the reactant complex in related processes.[^20^, ^28^]

An alternative pathway was considered starting from **C_0_** species. According to our calculations, the first step of this mechanism would be the substitution of the H_2_O molecule from the coordination sphere of the metal center by the H atom of the triethylsilane, leading to the **C_1_** intermediate, where an Ir−H bond is formed. This step occurs through the transition state **TS_1_**, where the originally coordinated H_2_O molecule is already displaced from the Ir coordination sphere (*d* Ir⋅⋅⋅O=3.98 Å), leaving a vacant position for the H transfer. This H transfer needs a nucleophilic attack of the back‐side water to happen, helped by the released front‐side water, which is oriented towards the triethylsilane moiety (*d* H⋅⋅⋅H=1.69 Å). Notice that this short H−H distance suggests that the H^∂+^−H^∂−^ interaction observed in **C_0_** is present in the **TS_1_** as well. The H transfer occurs at Ir⋅⋅⋅Si distance of 4.44 Å, being the hydrogen atom aligned midway the Ir and Si atoms, at 2.54 Å and 2.05 Å, respectively. Accordingly, in **TS_1_**, the Si atom is in a tbp coordination environment, as expected for the TS of a S_N_2 nucleophilic attack.

**C_1_** could be described as the adduct formed by the neutral dihydrido‐Ir compound and the tetracoordinated silylium cation formed, stabilized by the labile front‐side H_2_O molecule previously coordinated to the iridium center. This H_2_O molecule is H‐oriented towards the Ir−H moiety (*d* HO−H⋅⋅⋅H−Ir=1.71 Å). The partial atomic charges are calculated to be 0.518 e and −0.124 e. Both bond lengths and atomic charges are clear indications of the previously mentioned H^∂+^−H^∂−^ interaction.

The next step would be the formation of a pentacoordinated silylium cation [Et_3_Si(OH_2_)_2_]^+^,^[53]^ which is the consequence of the coordination of the front‐side H_2_O molecule to the silylium cation. This front side H_2_O is oriented like in **C_1_**, keeping the silylium cation close to the dihydrido‐iridium complex. The so‐formed intermediate is labelled as **C_2_**. In this complex, the electrostatic (H^∂+^−H^∂−^) interaction remains, as it may be concluded from the distance between the protic H−OH the hydride H−Ir, 1.57 Å, and the partial charges 0.547 and −0.118 e, respectively. From this intermediate, the protic H atom is ready to be transferred from the front‐side H_2_O to the dihydrido‐iridium complex through **TS_2_**, leading to the formation of an iridium‐H_2_ complex (**C_3_**) and Et_3_Si−OH species after the decomplexation of the back‐side H_2_O.

Finally, the decomplexation of dihydrogen would result in the formation of an iridium complex with a coordinative vacancy (intermediate **C_4_**), which would be occupied by a H_2_O molecule, leading the reactant complex **C_0_**, and closing the cycle. According to the calculated mechanism, the RDS would be the hydrogen transfer from the silicon to the iridium, through **TS_1_**, which is in good agreement with the experimental observations that suggest that Si−H activation is involved in the RDS.

In an effort to demonstrate theoretically the importance of hydrogen tunneling in the hydrolysis of triethylsilane catalyzed by **1**, theoretical KIEs have been calculated using Eyring and Bigelesein theories[^54^, ^55^, ^56^] (Table S.2, Supporting Information). These data (KIE_Eyring_=3.44; KIE_Bigelesein_=3.32), which does not include tunneling corrections, are very different from the experimental data (KIE_exp_=346). The difference between both values would be caused to the lack of tunneling corrections in the theoretical values, and are indicative of the importance of tunneling in these processes.^[57]^ Hence, the inclusion of tunneling effects in the theoretical calculations should increase the calculated KIE values. In this vein and in a simple way, one‐dimensional tunneling approaches, such as Wigner[^31^, ^58^, ^59^] and Bell's inverse parabola^[60]^ corrections to the KIE calculated by the Bigeleisen method have been implemented (Table S.2, Supporting Information).^[56]^ As expected, these two approaches underestimate the tunneling corrections,[^56^, ^61^] but still are able to increase slightly the calculated KIE.

Hence, more sophisticated approaches are mandatory to account for the expected tunneling effects to explain the large observed experimental KIE. In this vein, tunneling transmission probability for both hydrogen and deuterium were calculated via the one‐dimensional Wentzel‐Kramers‐Brillouin (WKB) semiclassical approach,^[62]^ which with a reasonable accuracy/time‐consuming balance provides a qualitatively accurate picture of the tunneling nature of the process. Thus, WKB method has been applied systematically, considering tunneling from all vibrational levels of H and D and their occupation probability using the Boltzmann distribution function (all the details are given in the Supporting Information), and the obtained results are collected in Table 3. At 298 K, the KIE including the tunneling effect is calculated to be 26.7. Now, the calculated KIE is one order of magnitude larger than the calculated with Wigner and/or Bell's tunneling corrections, being a clear indication of the importance of tunneling. Notice that even with this one‐dimensional approach, the importance of tunneling appears to be clear. In addition, the temperature effect in the calculated KIEs has been also calculated (Table 3). As may be observed, the predicted KIE values decrease as the temperature increases, as experimental ones. In addition, notice that the transmission probabilities calculated for both H and D increase with temperature, as expected, but those of D increase more rapidly, leading to smaller KIE values (see Supporting Information for a full discussion). Moreover, from predicted tunneling rate constants a A_H_/A_D_ ratio of 0.27 and *E* ${}_{a{}_{D}}$ −*E* ${}_{a{}_{H}}$ =11.4 kJ mol^−1^ were calculated, which are also indicative of tunnel effects.[^41^, ^42^] In this way, we have been able to demonstrate the participation of quantum tunneling by means of a one‐dimensional simple model.

**Table 3:** Calculated transmission probabilities for H, *τ*(H), and D, *τ*(D), and calculated theoretical and experimental KIEs at different temperatures, in K, errors in parenthesis.

*T* [K]	*τ*(H)	*τ*(D)	KIE_theo_	KIE_exp_
278	3.98×10^−9^	1.47×10^−10^	37.5	943(24)
283	4.81×10^−9^	1.95×10^−10^	34.3	821(11)
288	5.78×10^−9^	2.55×10^−10^	31.4	578(10)
293	6.92×10^−9^	3.32×10^−10^	28.9	484(7)
298	8.23×10^−9^	4.27×10^−10^	26.7	346(4)
303	9.73×10^−9^	5.45×10^−10^	24.7	299(7)

**Scheme 2:**
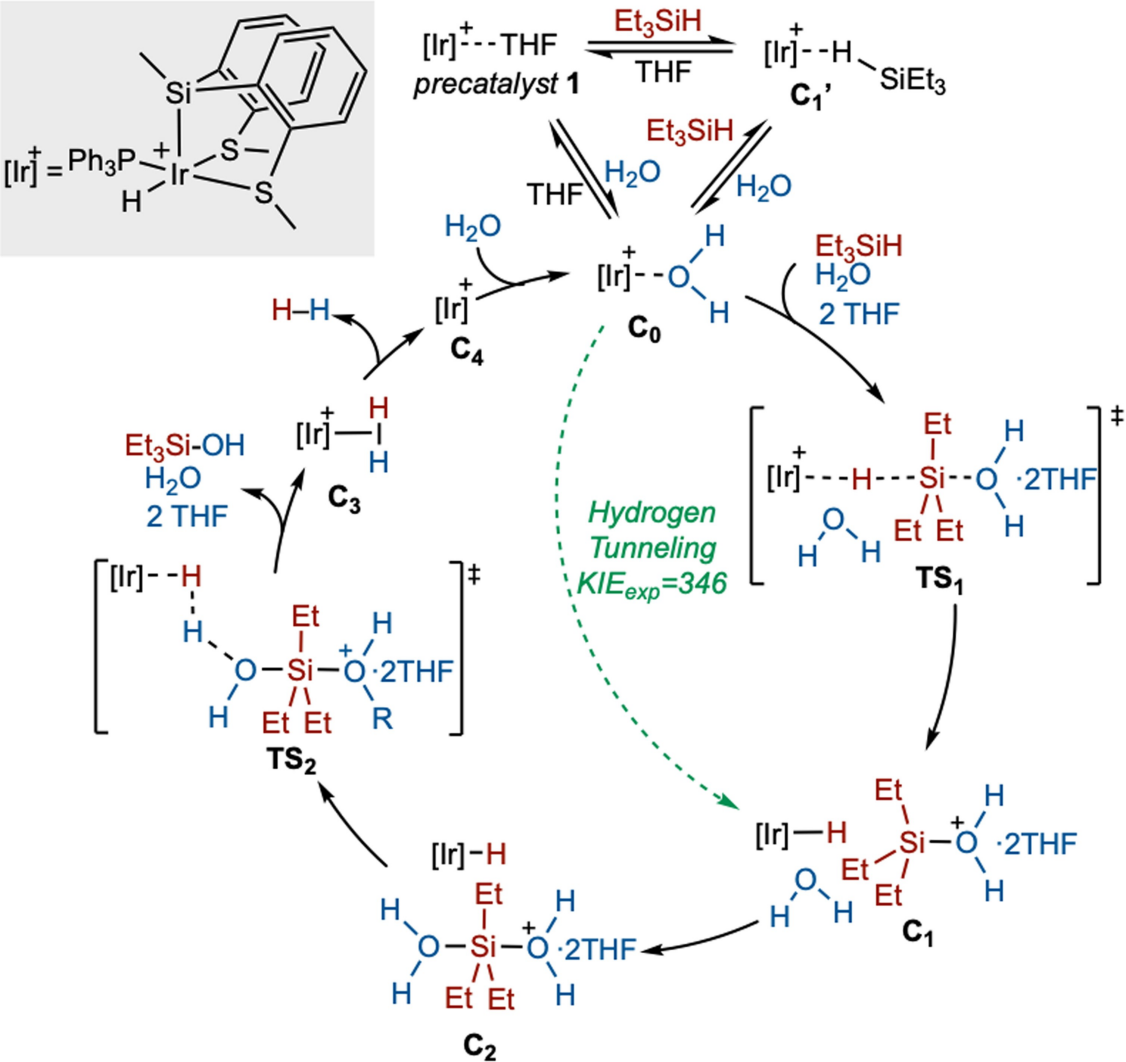
Catalytic cycle proposed for the hydrolysis of Et_3_SiH when **1** is used as catalyst.

Summarizing the computational results, they confirm the key role of hydrogen tunneling in the reaction mechanism, and provide a detailed mechanism for this reaction (Scheme 2). Slight differences with the accepted electrophilic mechanism for the hydrolysis of silanes have been found: i) the η^1^‐silane complex (**C_1_′** in Scheme 2) is not involved in the catalytic cycle; ii) the silylium cation is a pentacoordinated species ([Et_3_Si(OH_2_)_2_]^+^, **C_2_** in scheme 2). Scheme 2 shows the new alternative proposed mechanism for the hydrolysis of triethylsilane and the green dashed line represents the shortcut in the reaction pathway due to the quantum tunneling.

## Conclusion

The cationic hydrido‐silyl‐Ir^III^ complex **1**, behaved as an efficient precatalyst for the hydrolysis/alcoholysis of tertiary silanes leading to the release of one equivalent of dihydrogen and the corresponding silanols/silylethers. Kinetic studies on the process revealed *k*_Si‐H_/*k*_Si‐D_=346, which represents the largest one involving Si−H/D bonds observed up to date. This huge KIE in addition to the calculated Arrhenius factor and the difference in the observed activation energy are experimental indications of the participation of hydrogen quantum tunneling in the RDS of the process at room temperature. Detailed DFT calculation on the reaction mechanism allowed us to propose a slightly modified electrophilic mechanism for the reaction compared to the previously reported ones. In addition to this, theoretically calculated KIEs at different temperatures using the WKB one‐dimensional tunneling correction, support the importance of quantum tunneling. To the best of our knowledge, this is the first reported case of hydrosilane activation with the participation of quantum tunneling.

## Conflict of interest

The authors declare no conflict of interest.

1

## Supporting information

As a service to our authors and readers, this journal provides supporting information supplied by the authors. Such materials are peer reviewed and may be re‐organized for online delivery, but are not copy‐edited or typeset. Technical support issues arising from supporting information (other than missing files) should be addressed to the authors.

Supporting Information

## Data Availability

The data that support the findings of this study are available in the Supporting Information of this article.
