# Supplementary material for: Hydrogen Tunneling in Catalytic Hydrolysis and Alcoholysis of Silanes
Source: Angew Chem Int Ed Engl. 2022 Jul 29;61(36):e202204558. doi: 10.1002/anie.202204558 (PMC9545861; doi:10.1002/anie.202204558)
Supplement: Supplementary file 1 — Supporting Information [file ANIE-61-0-s001.pdf]

Angewandte Chemie
Eine Zeitschrift der Gesellschaft Deutscher Chemiker

Supporting Information

Hydrogen Tunneling in Catalytic Hydrolysis and Alcoholysis of Silanes

N. Almenara, M. A. Garralda, X. Lopez, J. M. Matxain, Z. Freixa*, M. A. Huertos**

Table of Contents

1. General considerations **S-3**
2. Catalytic experiments **S-3**
3. ^{1}H NMR characterization of silanols and silylethers **S-4**
4. Topographic maps of silanes **S-11**
5. Catalytic studies **S-11**
6. NMR studies **S-15**
7. Table of rate constants and $TOF_{1/2}$ **S-19**
8. Theoretical procedure **S-20**
9. Calculation of the theoretical Kinetic Isotope Effect by means of Eyring and Bigeleisen-Mayer approaches, including Wigner and Bell Inverse Parabola tunneling corrections **S-21**
10. Calculation of the KIE considering quantum tunneling by means of the WBK method **S-23**
11. References **S-28**
12. Cartesian Coordinates **S-29**

1. General Considerations

All manipulations, unless otherwise stated, were performed under an atmosphere of nitrogen, using standard Schlenk techniques. Glassware was oven-dried at 110 ºC overnight and flamed under vacuum prior to use. Dry and oxygen-free solvents were employed. {Ir[SiMe(o-$C_6H_4SMe)_2$](H)(PPh_3)(THF)}[$BAr^F{}_4$] (**1**), {IrCl[SiMe(o-$C_6H_4SMe)_2$](H)(PPh_3)} (**2**), Me_2PhSiD and Ph_3SiD were prepared as previously described.[1] Et_3SiH, Et_3SiD, Me_2PhSiH, $MePh_2SiH$ and Ph_3SiH were purchased from Merck and used without previous purification. MeOH, EtOH and ^{i}PrOH were used dry and oxygen-free. NMR spectra were recorded on Bruker AVD 400 MHz spectrometer. ^{1}H spectra were referenced to the residual solvent signals. Chemical shifts are quoted in ppm and coupling constants in Hz.

2. Catalytic experiments

A closed reaction vessel equipped with a pressure transducer (Manonthemoon kinetic kit X102)[2] was immersed in a thermostated ethylene glycol/water bath and charged with the catalyst (**1**, 4.2 mg, 0.0025 mmol; **2**, 1.9 mg, 0.0025 mmol; and [Ir(cod)Cl]$_2$, 0.85 mg, 0.00125 mmol) in 1 mL of distilled THF and H_2O or alcohol (2.5 mmol). Once the pressure of the system was stabilized, the silane (0.25 mmol) was added, which was considered initial reaction time. The solution was left stirring until the pressure stabilized again, which was indicative that the reaction ended. Then, the reaction mixture was filtered through a small silica pad eluting with pentane to remove the catalyst, and the solvent was removed under vacuum. The residue was analyzed by ^{1}H NMR in $CDCl_3$. The quantity of gas evolved was calculated from the measured pressure inside the reaction vessel following the ideal gases law equation (reactor volume 13.2 mL).

3. ^{1}H NMR characterization of silanols and silylethers

Triethylsilanol

^{1}H NMR (400 MHz, $CDCl_3$): δ 2.33 (s, 1H, Si-OH), 0.98 (t, $J_{H\text{-}H}$ = 8 Hz, 9H, $-CH_3$), 0.60 (q, $J_{H\text{-}H}$ = 8 Hz, 6H, Si-CH_2).
^{1}H-^{29}Si HMBC NMR (400 MHz, $CDCl_3$): δ(^{29}Si) 18.8 ppm.

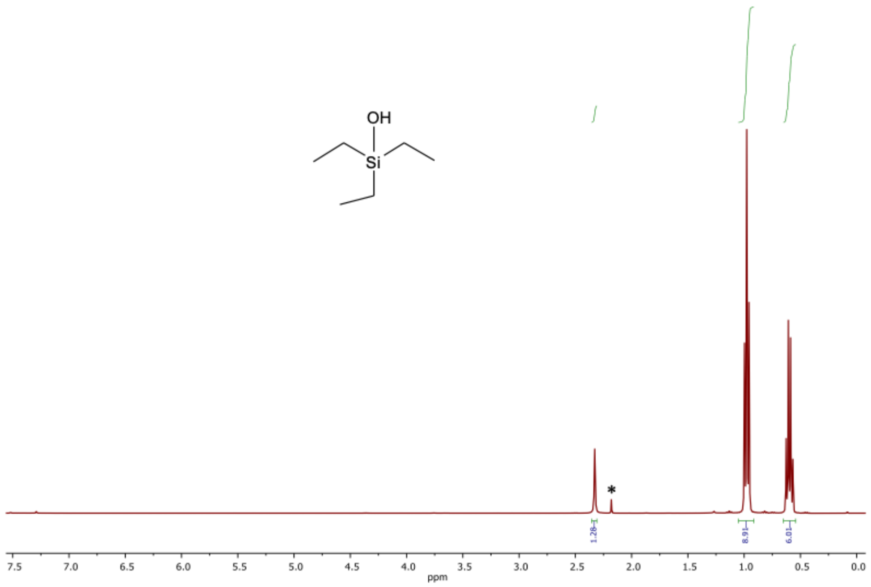

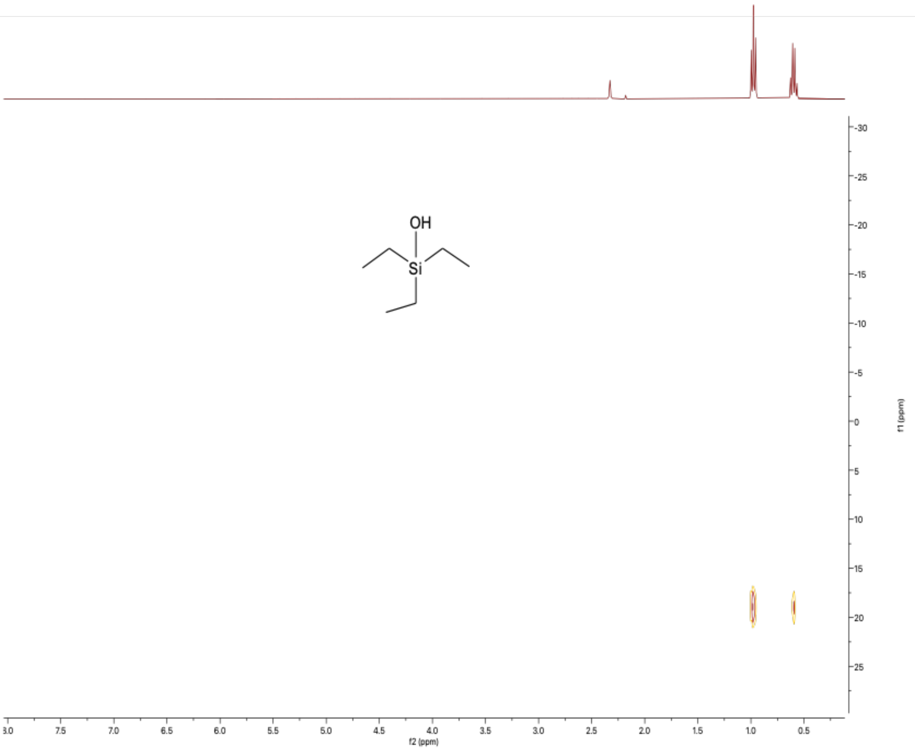

Figure S1. ^{1}H NMR spectrum of triethylsilanol. (*acetone) (top). ^{1}H-^{29}Si HMBC NMR spectrum of triethylsilanol (bottom).

Dimethylphenylsilanol

^{1}H NMR (400 MHz, $CDCl_3$): δ 7.63 (m, 2H, aromatics), 7.42 (m, 3H, aromatics), 2.52 (s, 1H, Si-OH), 0.43 (s, 6H, Si-CH_3).

^{1}H-^{29}Si HMBC NMR (400 MHz, $CDCl_3$): δ(^{29}Si) 6.9 ppm.

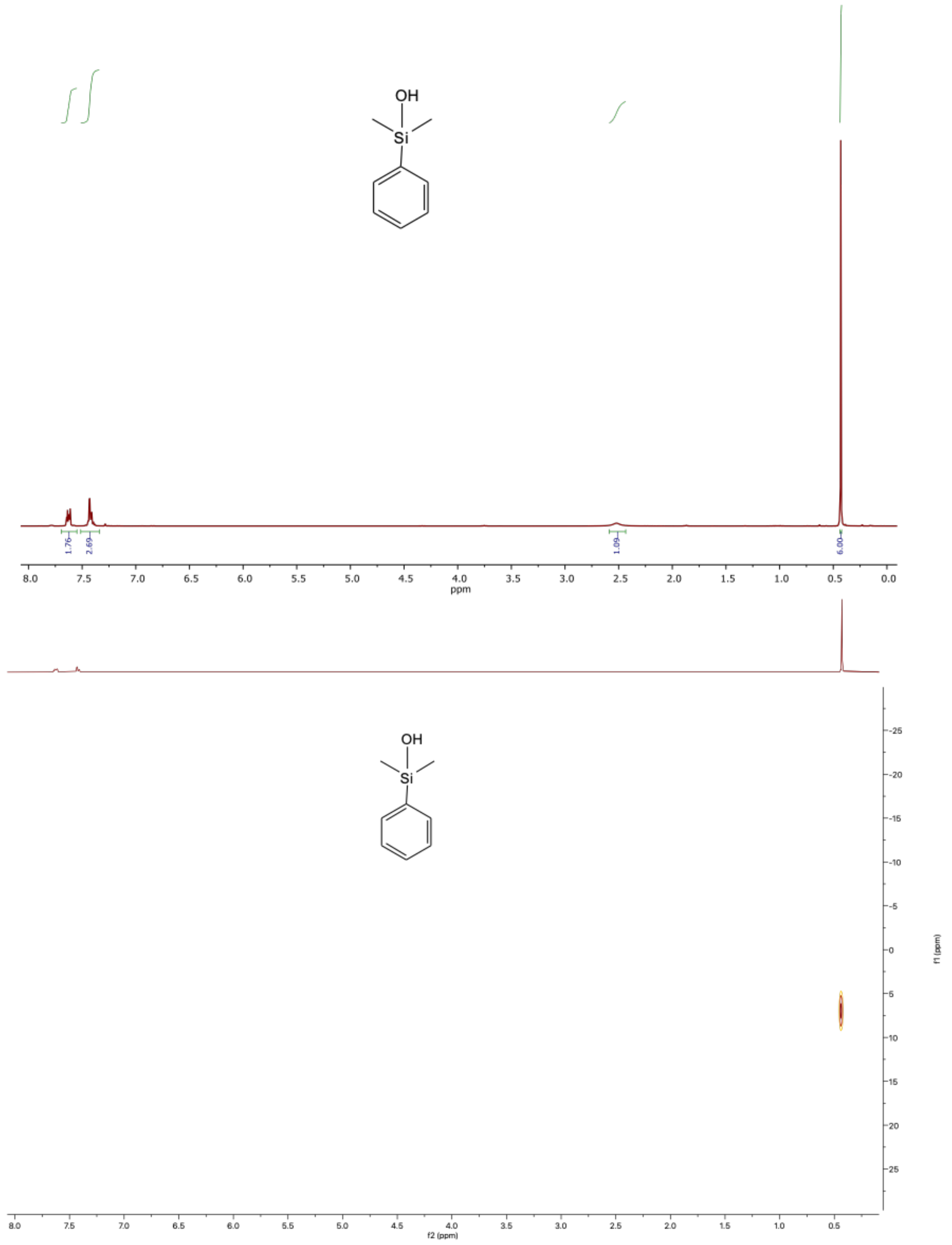

Figure S2. ^{1}H NMR spectrum of dimethylphenylsilanol (top). ^{1}H-^{29}Si HMBC NMR spectrum of dimethylphenylsilanol (bottom).

Methyldiphenylsilanol

^{1}H NMR (400 MHz, $CDCl_3$): δ 7.65 (m, 4H, aromatics), 7.44 (m, 6H, aromatics), 2.71 (s, 1H, Si-OH), 0.70 (s, 3H, Si-CH_3).
^{1}H-^{29}Si HMBC NMR (400 MHz, $CDCl_3$): δ(^{29}Si) -2.8 ppm.

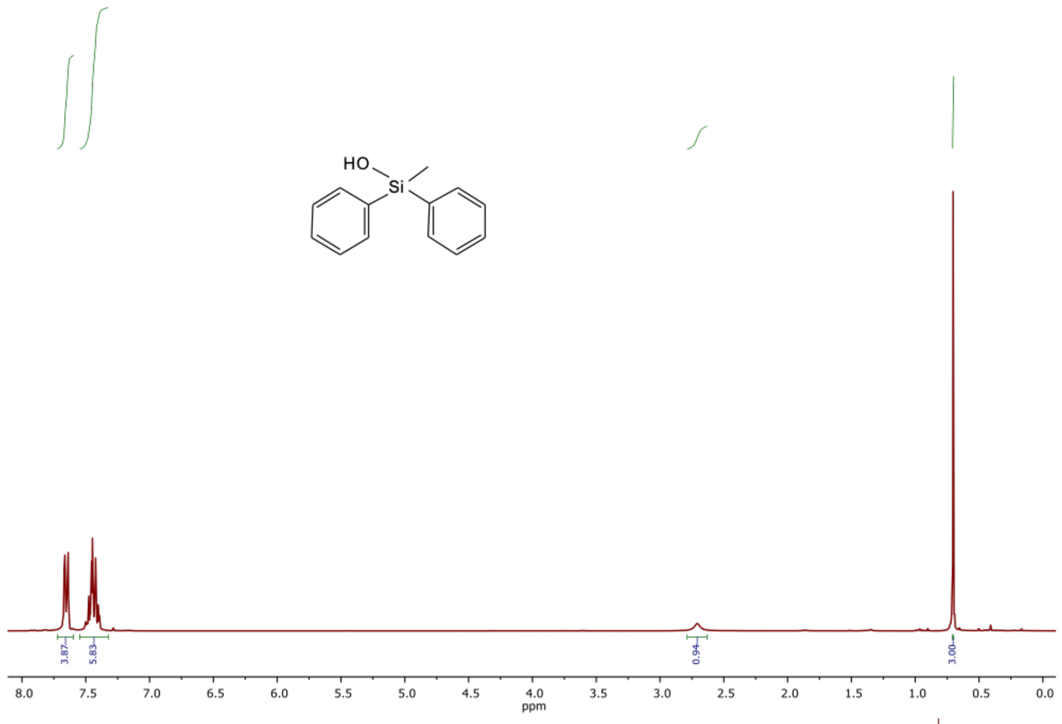

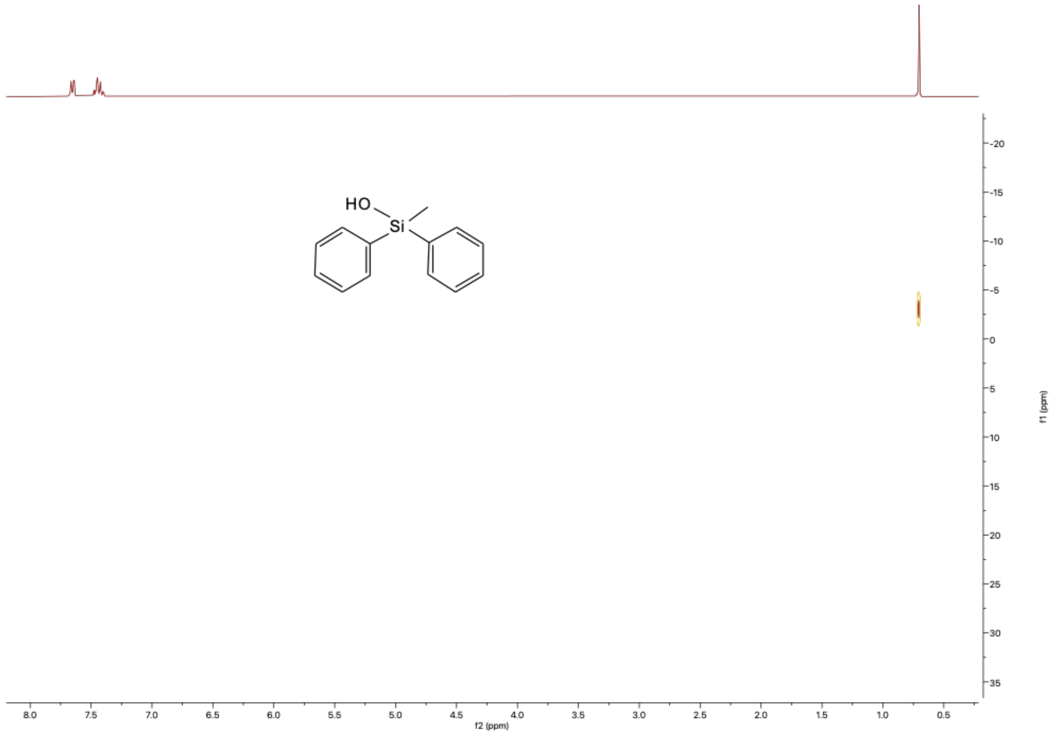

Figure S3. ^{1}H NMR spectrum of methyldiphenylsilanol (top). ^{1}H-^{29}Si HMBC NMR spectrum of methyldiphenylsilanol (bottom).

Triphenylsilanol

^{1}H NMR (400 MHz, $CDCl_3$): δ 7.67 (m, 6H, aromatics), 7.43 (m, 9H, aromatics), 2.49 (s, 1H, Si-OH).
^{1}H-^{29}Si HMBC NMR (400 MHz, $CDCl_3$): δ(^{29}Si) -12.2 ppm.

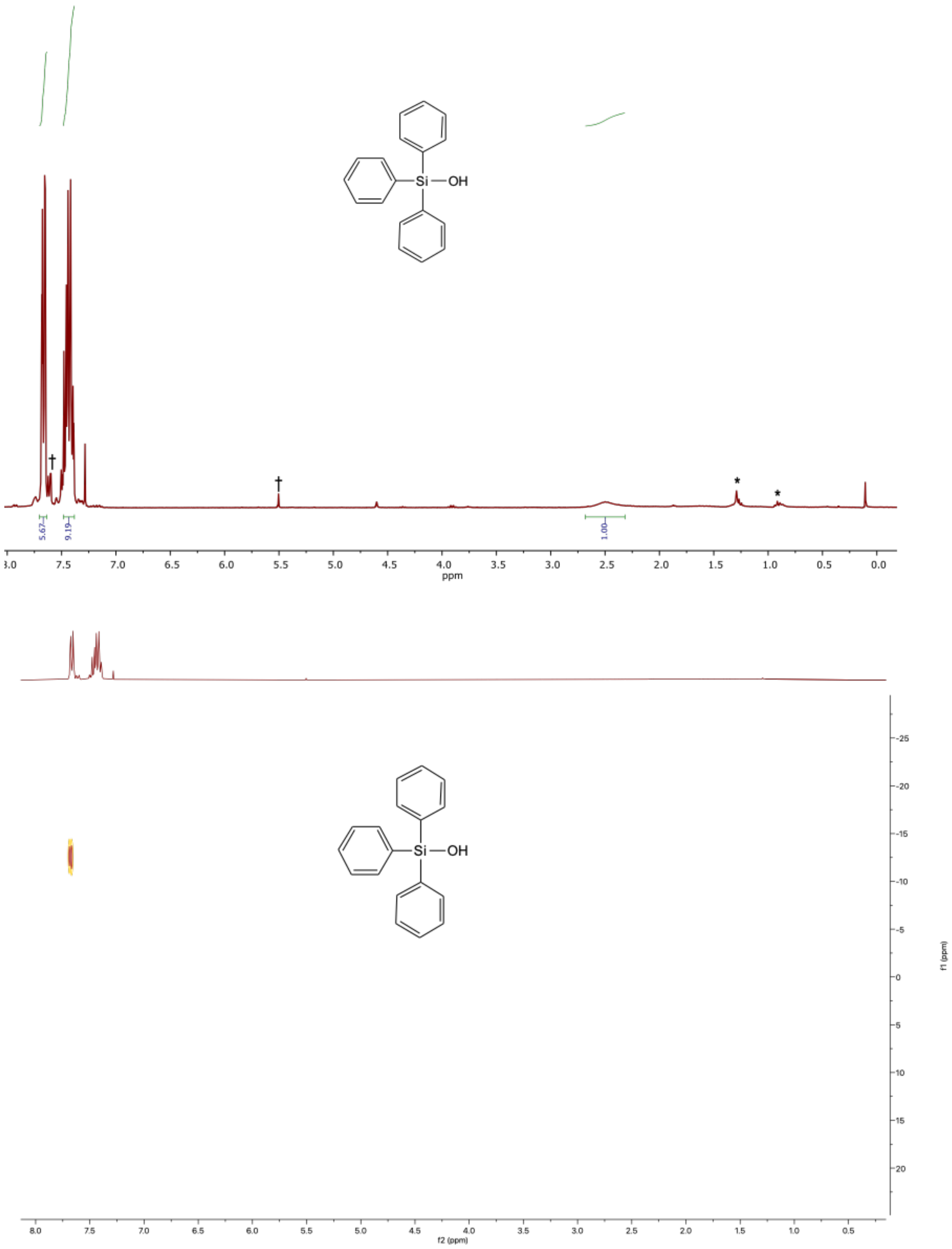

Figure S4. ^{1}H NMR spectrum of triphenylsilanol. (*pentane, † PPh_3SiH) (top). ^{1}H-^{29}Si HMBC NMR spectrum of triphenylsilanol (bottom).

Triethylmethoxysilane

^{1}H NMR (400 MHz, $CDCl_3$): δ 3.50 (s, 3H, OCH_3), 0.99 (t, $J_{H\text{-}H}$ = 8 Hz, 9H), 0.64 (q, $J_{H\text{-}H}$ = 8 Hz, 6H).

^{1}H-^{29}Si HMBC NMR (400 MHz, $CDCl_3$): δ(^{29}Si) 19.9 ppm.

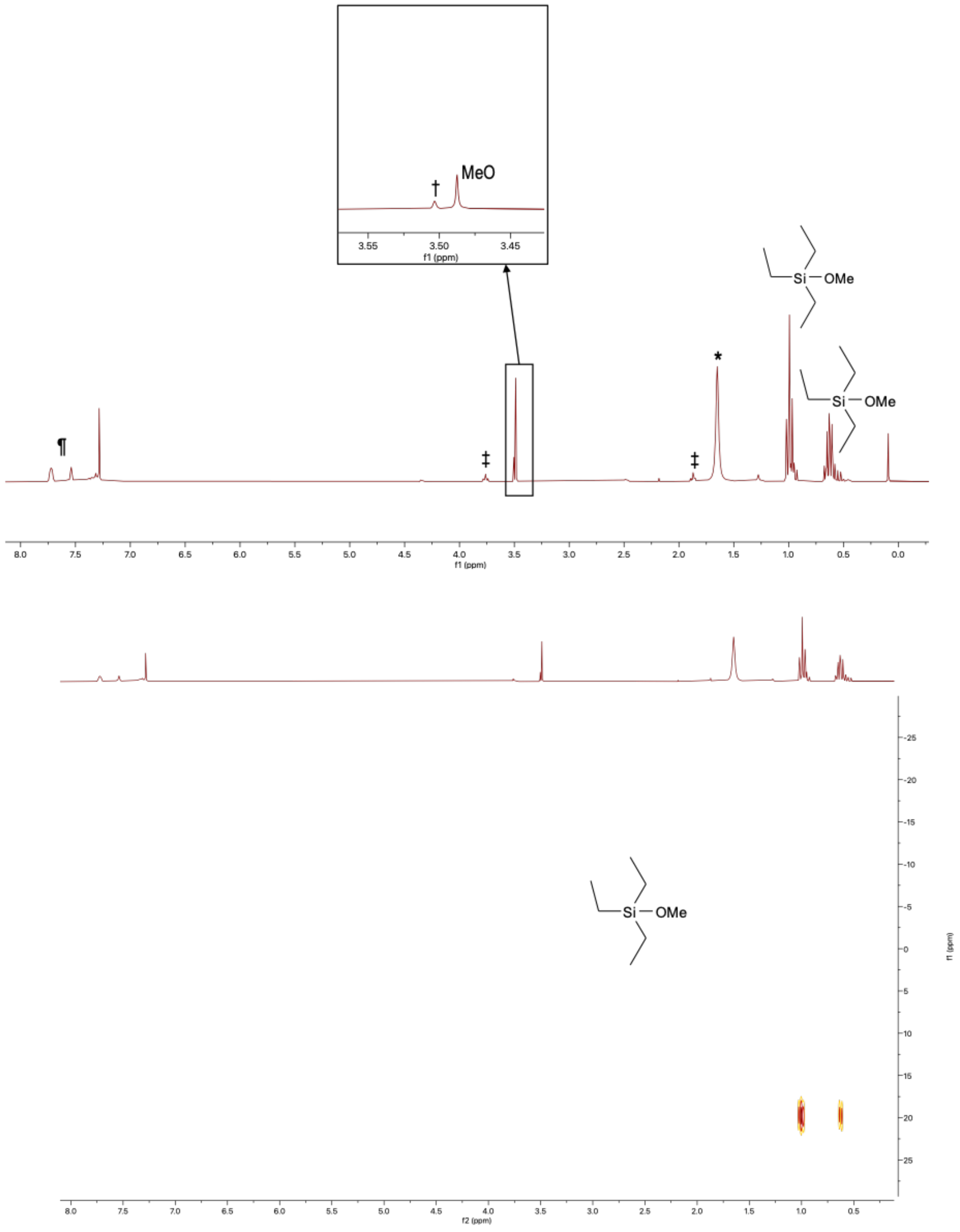

Figure S5. ^{1}H NMR spectrum of triethylmethoxysilane. (†MeOH, *water, ‡THF, ¶[BAr$^{F}_{4}$] from precatalyst **1**) (top). ^{1}H-^{29}Si HMBC NMR spectrum of triethylmethoxysilane (bottom).

Triethylethoxysilane

^{1}H NMR (400 MHz, $CDCl_3$): δ 3.69 (q, $J_{H\text{-}H}$ = 7 Hz, 2H, OCH_2CH_3), 1.19 (t, $J_{H\text{-}H}$ = 7 Hz, 3H, OCH_2CH_3), 0.97 (t, $J_{H\text{-}H}$ = 8 Hz, 9H), 0.59 (q, $J_{H\text{-}H}$ = 8 Hz, 6H).

^{1}H-^{29}Si HMBC NMR (400 MHz, $CDCl_3$): δ(^{29}Si) 18.9 ppm.

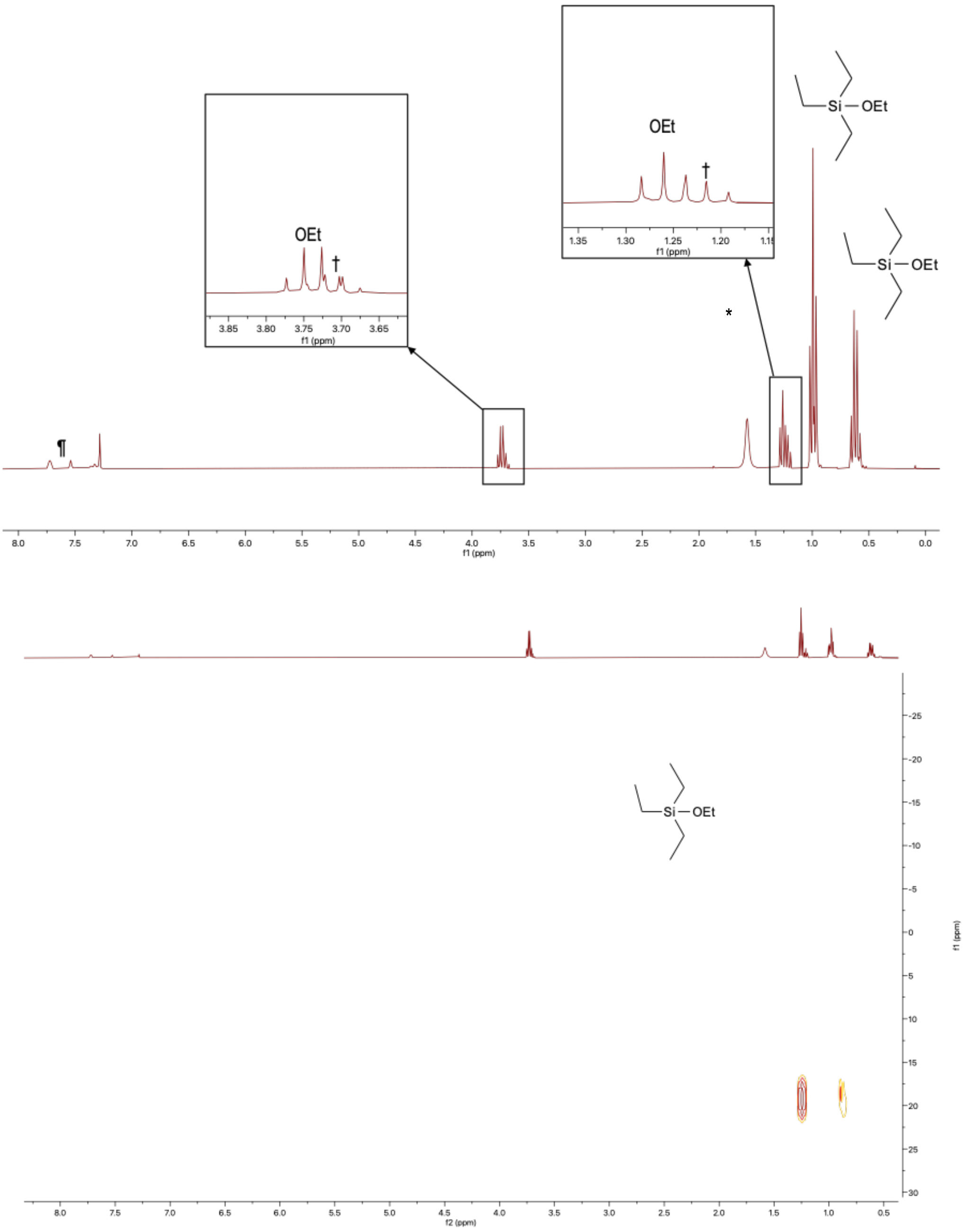

Figure S6. ^{1}H NMR spectrum of triethylethoxysilane. (†EtOH, *water, ¶[BAr^{F}_4] from precatalyst **1**) (top). ^{1}H-^{29}Si HMBC NMR spectrum of triethylethoxysilane (bottom).

Triethylisopropoxysilane

^{1}H NMR (400 MHz, CDCl$_3$): δ 3.99 (sep, $^3J_{H\text{-}H}$ = 6 Hz, 1H, CH ^{i}PrO), 1.15 (d, $^3J_{H\text{-}H}$ = 6 Hz, 6H, CH$_3$ ^{i}PrO), 0.97 (t, $^3J_{H\text{-}H}$ = 8 Hz, 9H), 0.60 (q, $^3J_{H\text{-}H}$ = 8 Hz, 6H).

^{1}H-^{29}Si HMBC NMR (400 MHz, CDCl$_3$): δ(^{29}Si) 17.1 ppm.

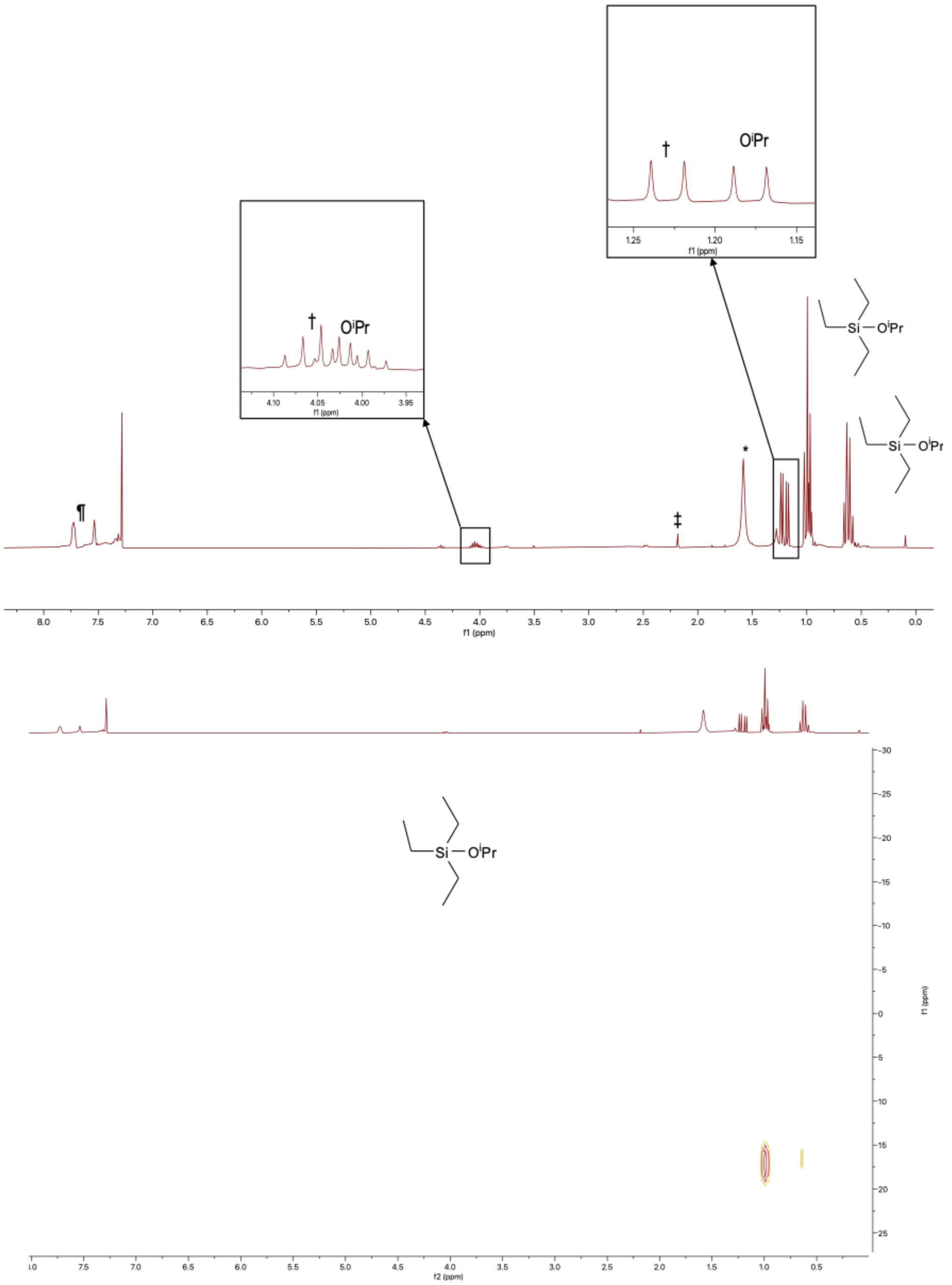

Figure S7. ^{1}H NMR spectrum of triethylisopropoxysilane. (^{†i}PrOH, *water, ‡acetone, ¶[BAr$^F{}_4$] from precatalyst **1**) (top). ^{1}H-^{29}Si HMBC NMR spectrum of triethylisopropoxysilane (bottom).

4. Topographic maps of silanes

Steric maps were evaluated with the SambVca 2.0 package.The radius of the sphere around the centre atom was set to: 3.5 Å or 5 Å, distance from the centre of the sphere: 2.26 Å, mesh spacing: 0.1 Å, H atoms omitted and atom radii: Bondi radii scaled by 1.17, as recommended by Cavallo.[3]

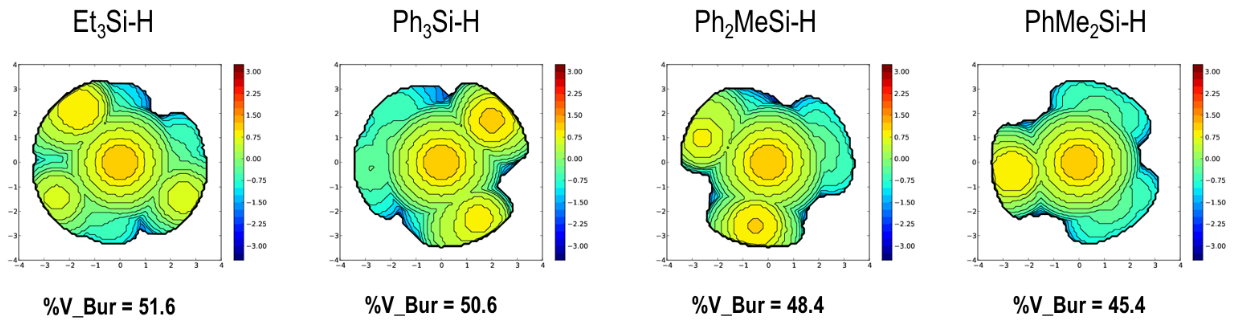

Figure S8. Topographic maps and percent buried volume for Et_3SiH, Me_2PhSiH, $MePh_2SiH$ and Ph_3SiH.

5. Catalytic studies

Hydrolysis of silanes using 1

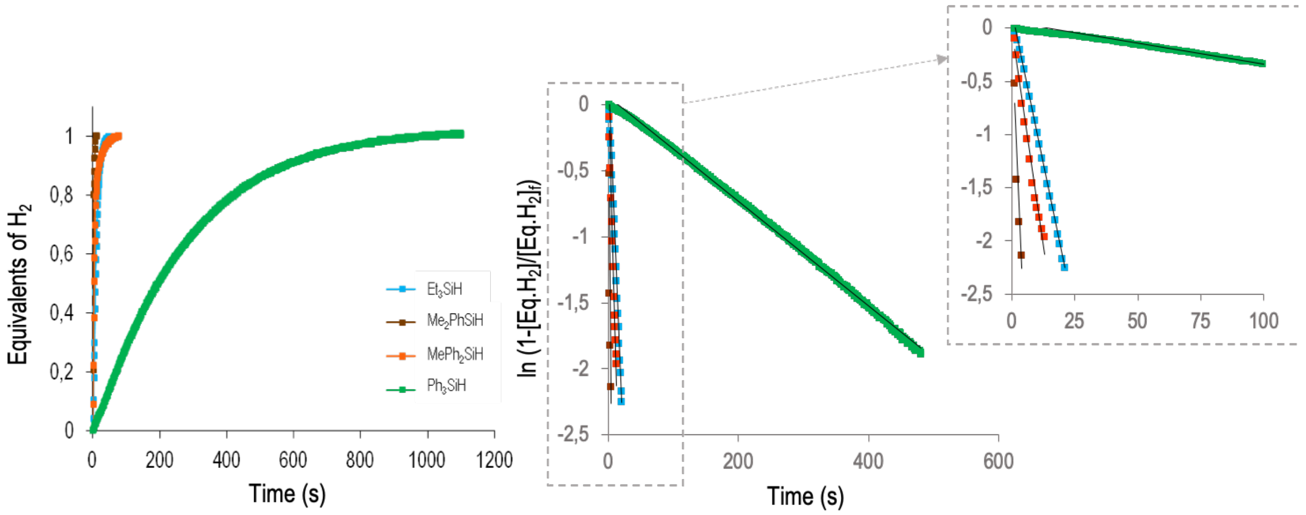

Figure S9. Reaction profiles (equivalents of H_2 *vs* time) and first-order plots for the hydrolysis of different silanes using **1** (1 mol %) as precatalyst at 25 ºC.

Alcoholysis of Et_3SiH using 1

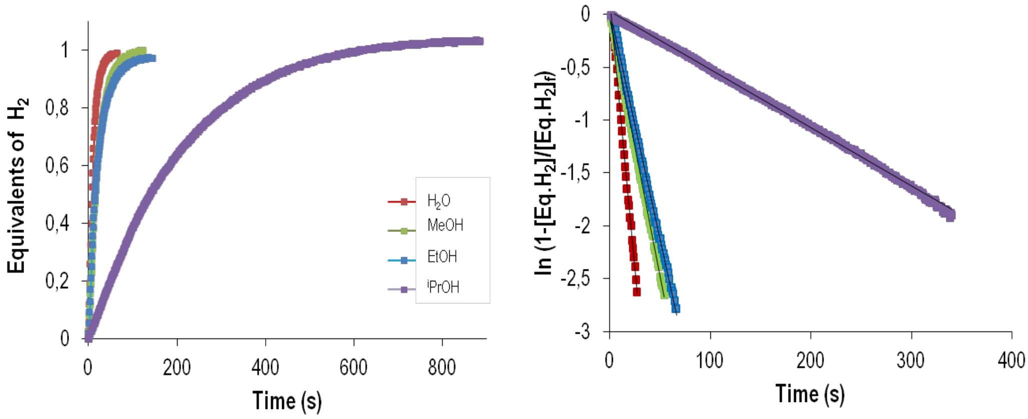

Figure S10. Reaction profiles (equivalents of H_2 *vs* time) and first-order plots for the alcoholysis of Et_3SiH using **1** (1 mol %) as precatalyst at 25 ºC.

Hydrolysis of Et_3SiH using different concentrations of catalyst, water and silane.

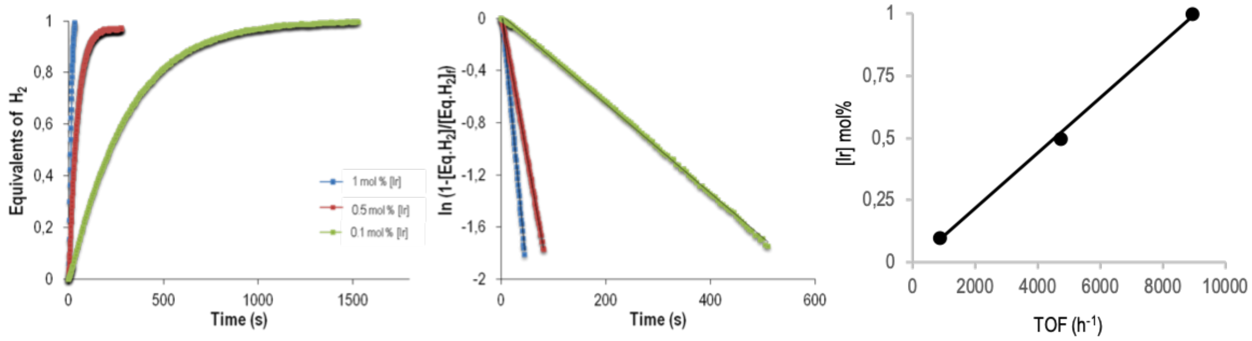

Figure S11. Reaction profiles (equivalents of H_2 *vs* time) obtained for the hydrolysis of Et_3SiH using different concentrations of precatalyst **1** at 25 ºC and TOF *vs* catalyst concentration plot.

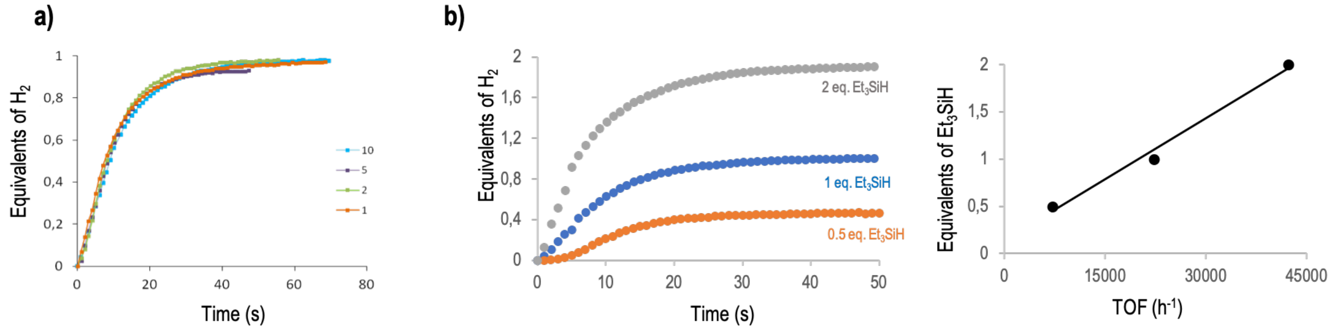

Figure S12. a) Reaction profiles (equivalents of H_2 *vs* time) obtained for the hydrolysis of Et_3SiH with precatalyst **1** (1 mol %) using different equivalents of water at 25 ºC. b) Reaction profiles (equivalents of H_2 *vs* time) obtained for the hydrolysis of Et_3SiH with precatalyst **1** (1 mol %) using different equivalents of silane at 25 ºC and TOF *vs* equivalents of silane plot.

Thermodynamic study of the hydrolysis of Et_3SiH using 1 as catalyst

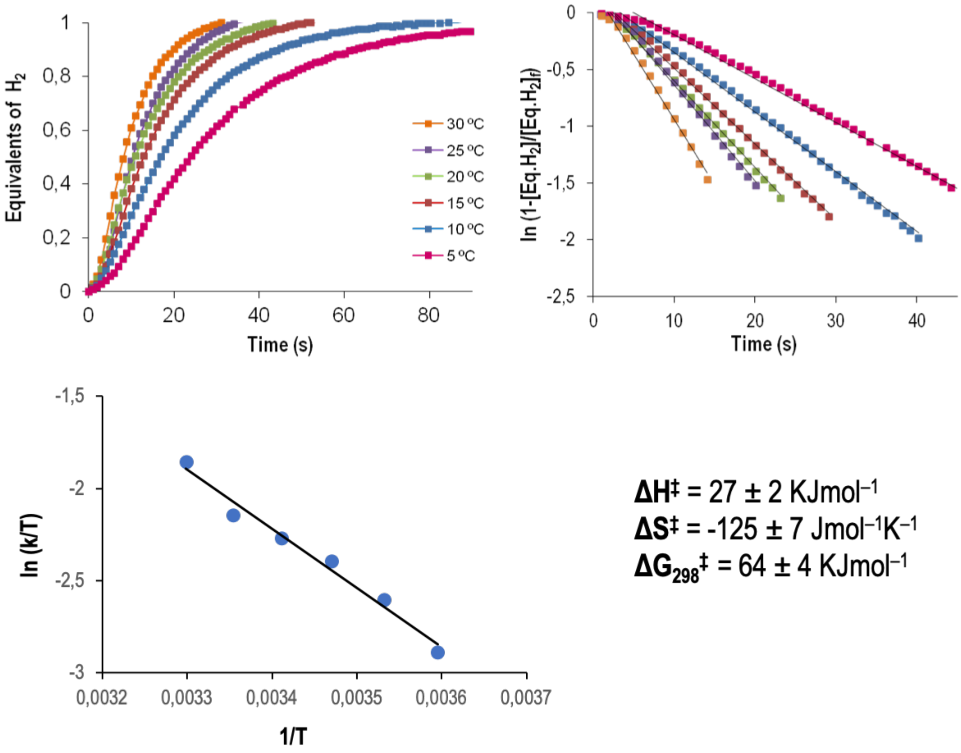

Figure S13. Reaction profiles (equivalents of H_2 *vs* time) and first-order plots for the hydrolysis of Et_3SiH with precatalyst **1** (1 mol %) at different temperatures (5-30 ºC).

Kinetic isotope effect studies in the hydrolysis and methanolysis of Silanes using 1 as catalyst

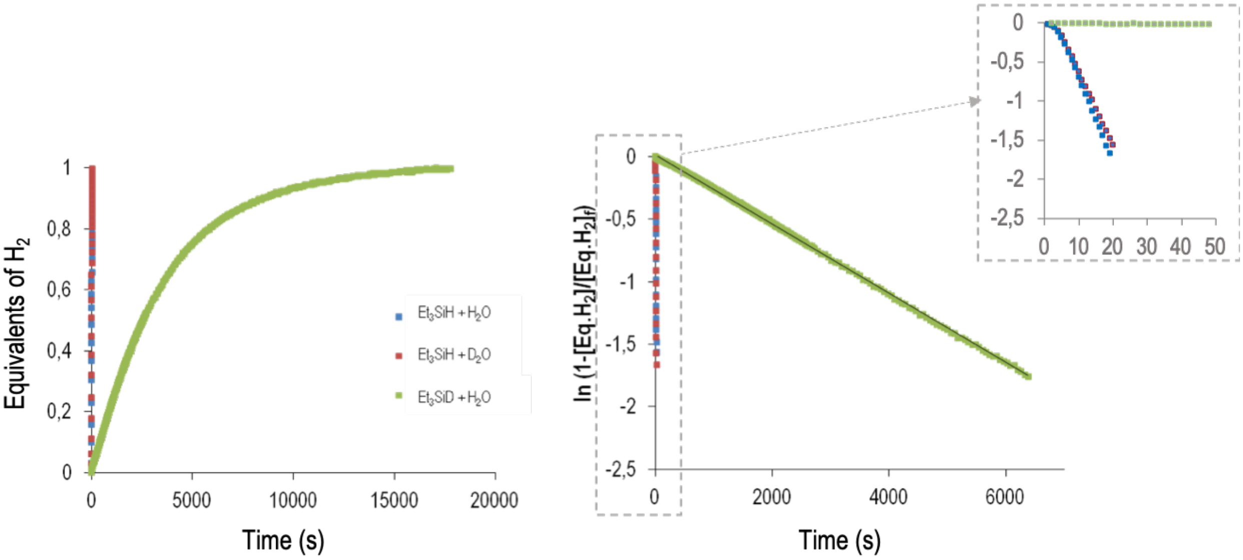

Figure S14. KIE studies: Reaction profiles and first order plots for the hydrolysis of Et_3SiH with precatalyst **1** (1 mol %) at 25 ºC.

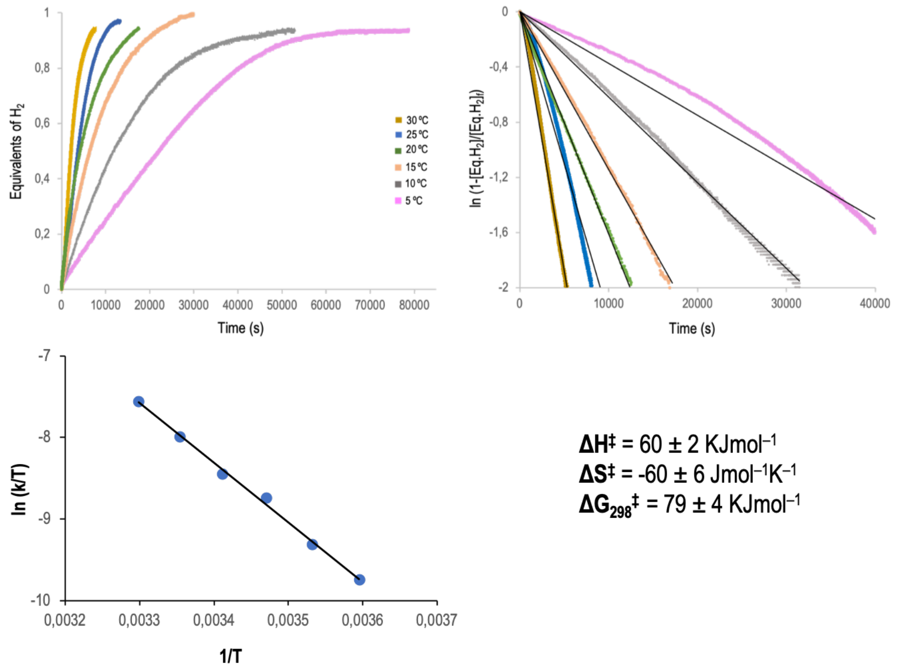

$\Delta H^{\ddagger}$ = 60 ± 2 KJmol^{-1}
$\Delta S^{\ddagger}$ = -60 ± 6 Jmol^{-1}K^{-1}
$\Delta G_{298}^{\ddagger}$ = 79 ± 4 KJmol^{-1}

Figure S15. Reaction profiles and first-order plots for the hydrolysis of Et_3SiD with precatalyst **1** (1 mol %). Temperature range 5–30 ºC.

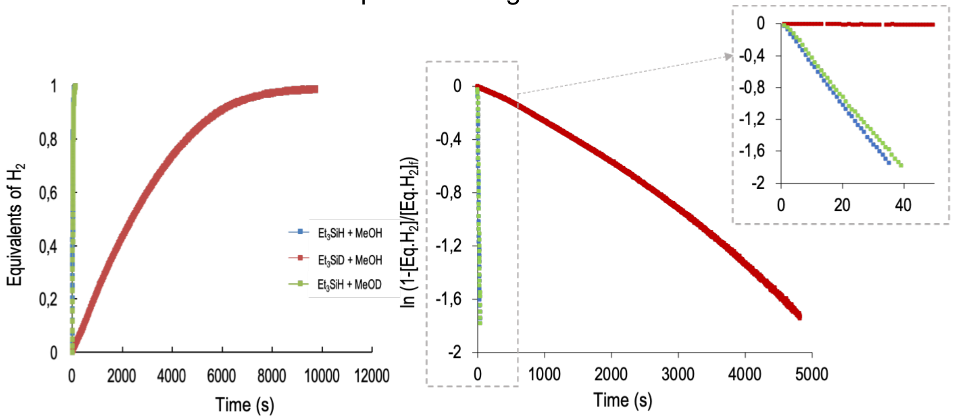

Figure S16. KIE studies: Reaction profiles and first-order plots for the methanolysis of Et_3SiH with precatalyst **1** (1 mol %) at 25 ºC.

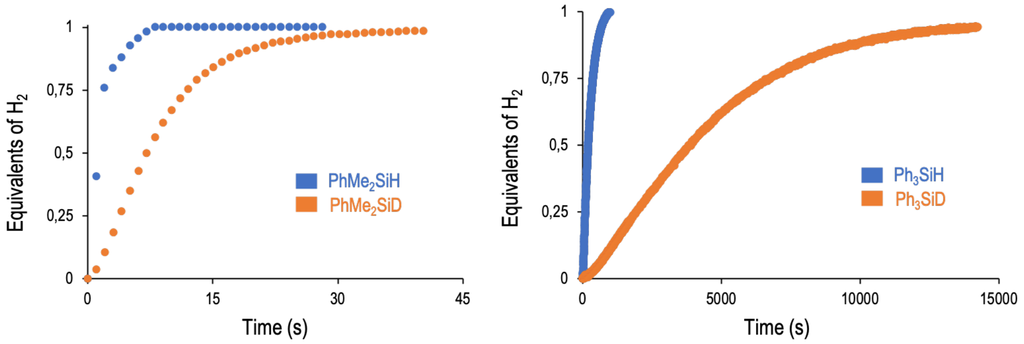

$k_{PhMe2SiH}$= 219.2(25) s^{-1} M^{-1}
$k_{PhMe2SiD}$= 50.6(15) s^{-1} M^{-1}
KIE= 4.3(5)

k_{Ph3SiH}= 1.58(1) s^{-1} M^{-1}
k_{Ph3SiD}= 0.094(1) s^{-1} M^{-1}
KIE= 16.8 (2)

Figure S17. KIE studies: Reaction profiles the hydrolysis of $PhMe_2SiH$ (left) and Ph_3SiH (right) with precatalyst **1** (1 mol %) at 25 ºC.

Kinetic isotope effect study in the hydrolysis of Et_3SiH using $[Ir(cod)Cl]_2$ as catalyst

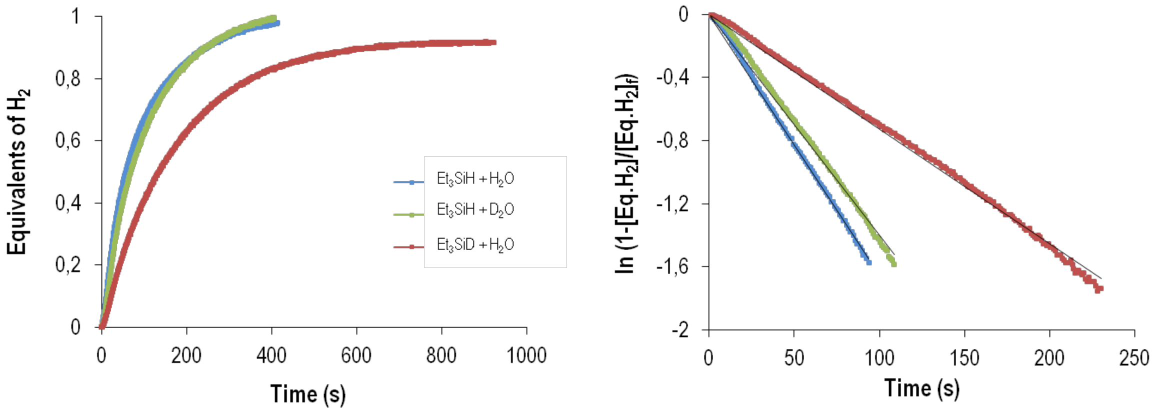

Figure S18. Reaction profiles and first-order plots for the KIE studies in the hydrolysis of Et_3SiH with $[Ir(cod)Cl]_2$ at 25 ºC.

6. NMR studies

NMR studies of formation of H_2/HD in the hydrolysis of trithylsilane: High pressure NMR tube was charged with 0.00125 mmol of **1** (2.1 mg), 0.125 mmol of triethylsilane (Et_3SiH, 19.5 μL; Et_3SiD 20 μL), 1.25 mmol of water (H_20, 22 μL; D_2O 23 μL) and THF-d_8 (0.5 mL). ^{1}H NMR spectra were performed after 30 minutes (full conversion). Spectra shown in Figure S19 have been normalized to Et_3Si-OH signals in order to estimate the approximate amount of gas (H_2, HD and D_2) formed. We have not been able to obtain useful ^{2}H NMR spectra since D_2O and D_2 signals are overlapped.

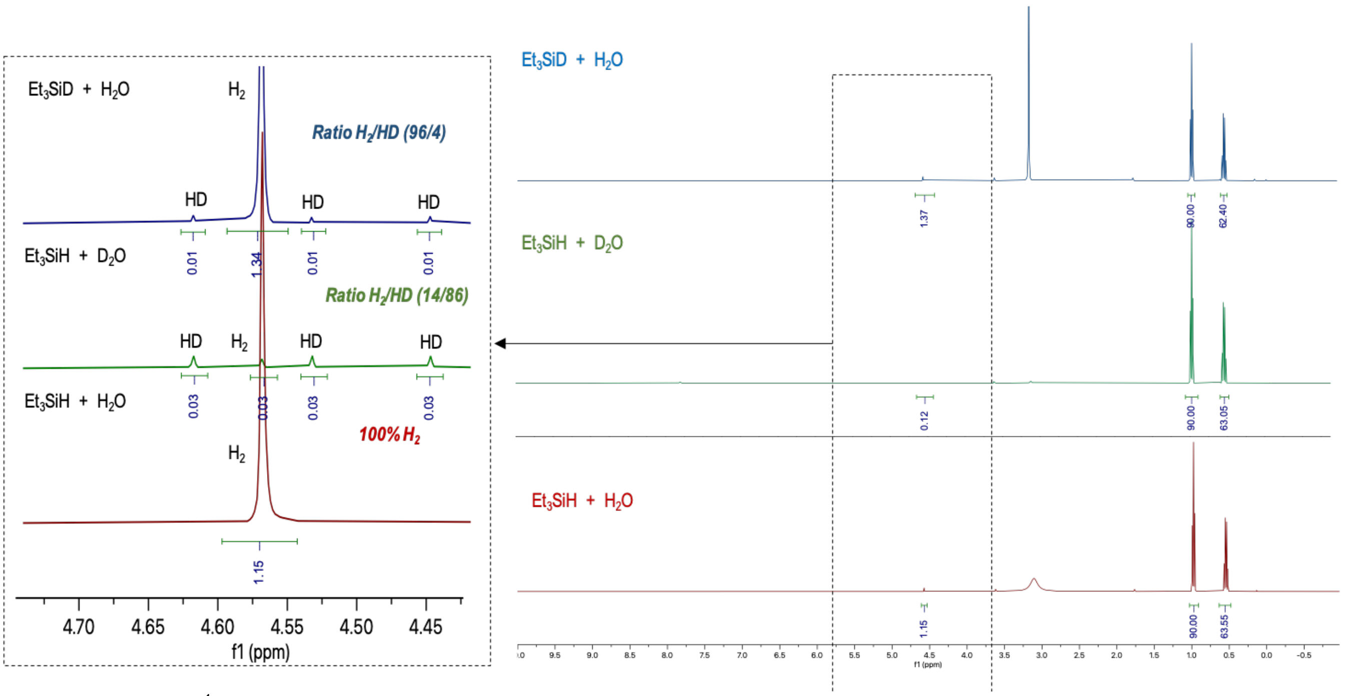

Figure S19. ^{1}H NMR spectra of the catalytic hydrolysis of triethylsilane. $Et_3SiH + H_2O$ (red). $Et_3SiH + D_2O$ (green). $Et_3SiD + H_2O$ (blue).

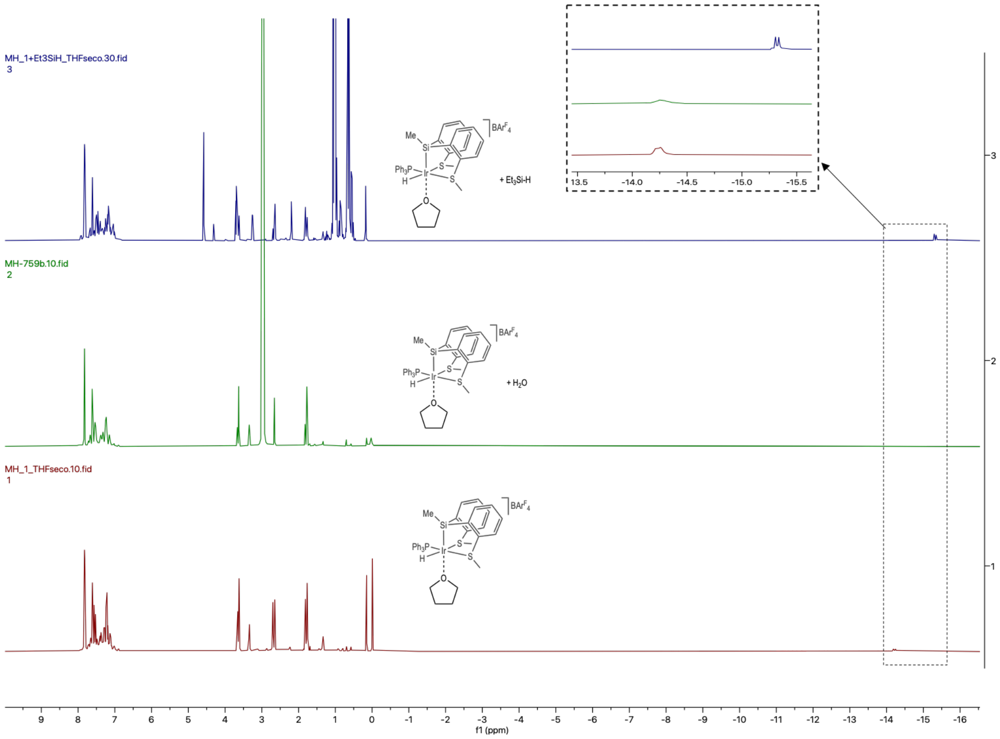

Figure S20. ^{1}H NMR spectra of **1** (10 mg, $5.95 \cdot 10^{-3}$ mmol) dissolved in 0.5 mL of THF-d_8 (red). **1** + 100 eq.H_2O in THF-d_8 (green). **1** + 10 eq. Et_3SiH in THF-d_8 (blue)

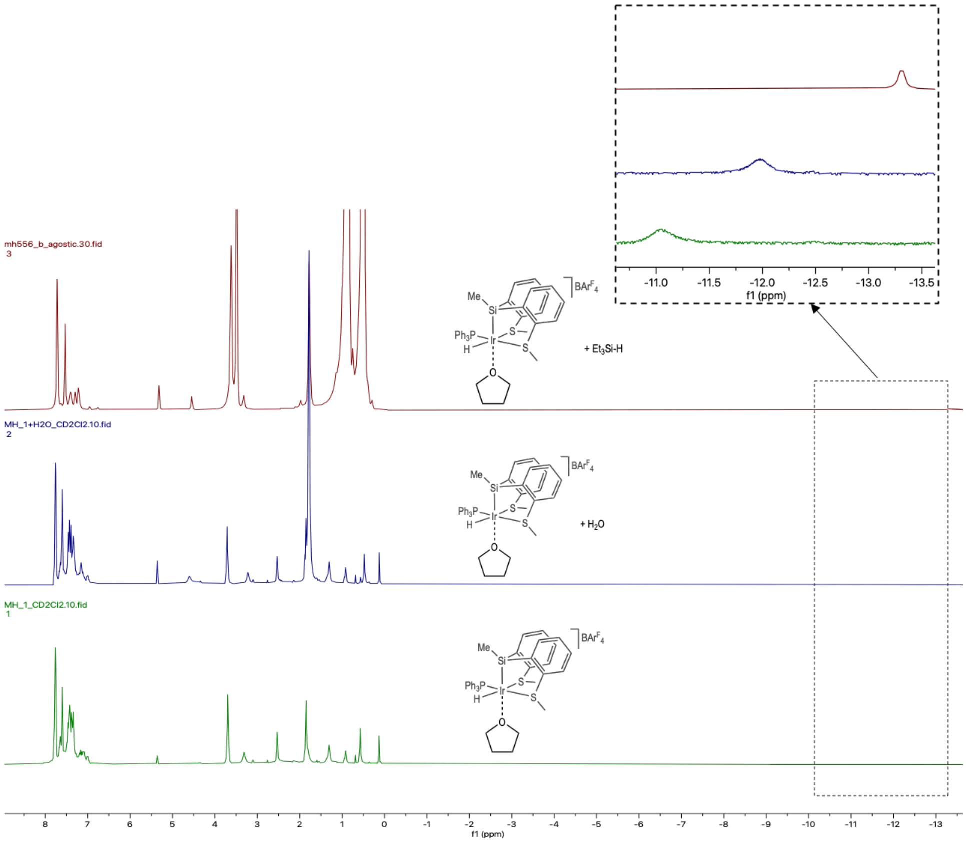

Figure S21. ^{1}H NMR spectra of **1** (10 mg, $5.95 \cdot 10^{-3}$ mmol) dissolved in 0.5 mL of CD_2Cl_2 (red). **1** + 100 eq.H_2O in CD_2Cl_2 (green). **1** + 10 eq. Et_3SiH in CD_2Cl_2 (green).

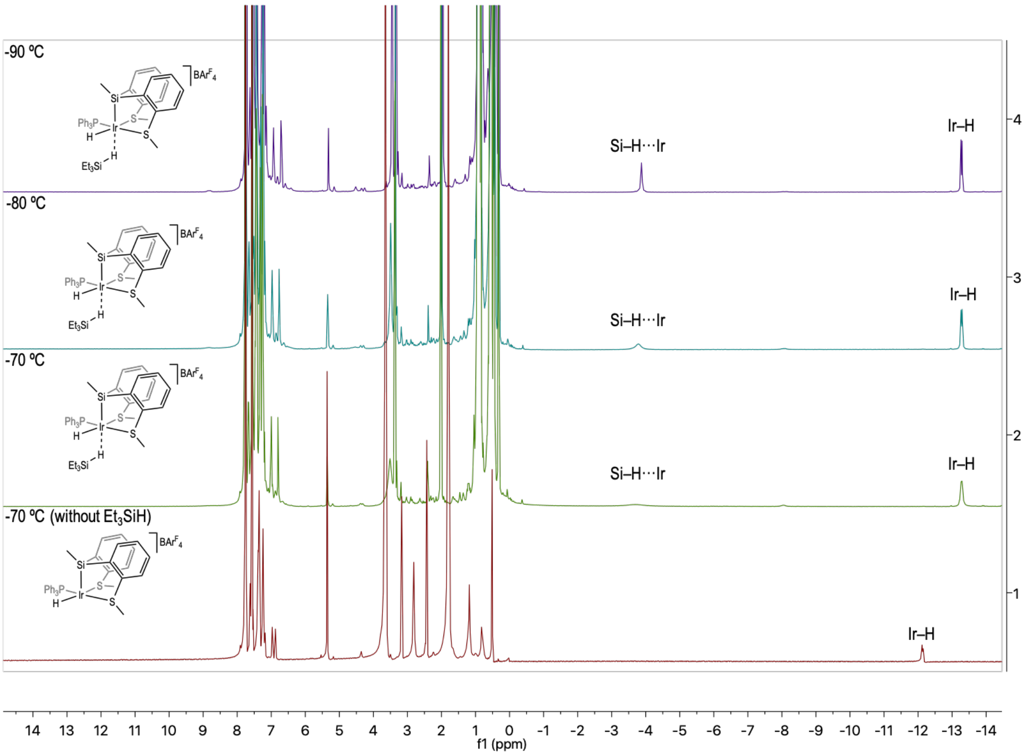

Figure S22. ^{1}H NMR spectra of **1** (10 mg, $5.95 \cdot 10^{-3}$ mmol) dissolved in 0.5 mL of CD_2Cl_2 before (red) and after adding 10 eq. of Et_3SiH at -70 ºC (green), -80ºC (blue) and -90ºC (purple).

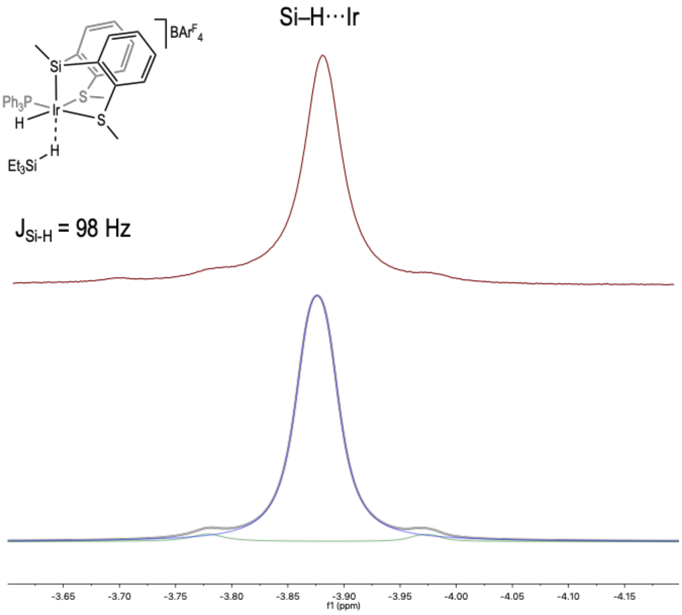

Figure S23. Si-H···Ir region of the ^{1}H NMR spectra of **3** at -90ºC (top) and peaks deconvolution (bottom).

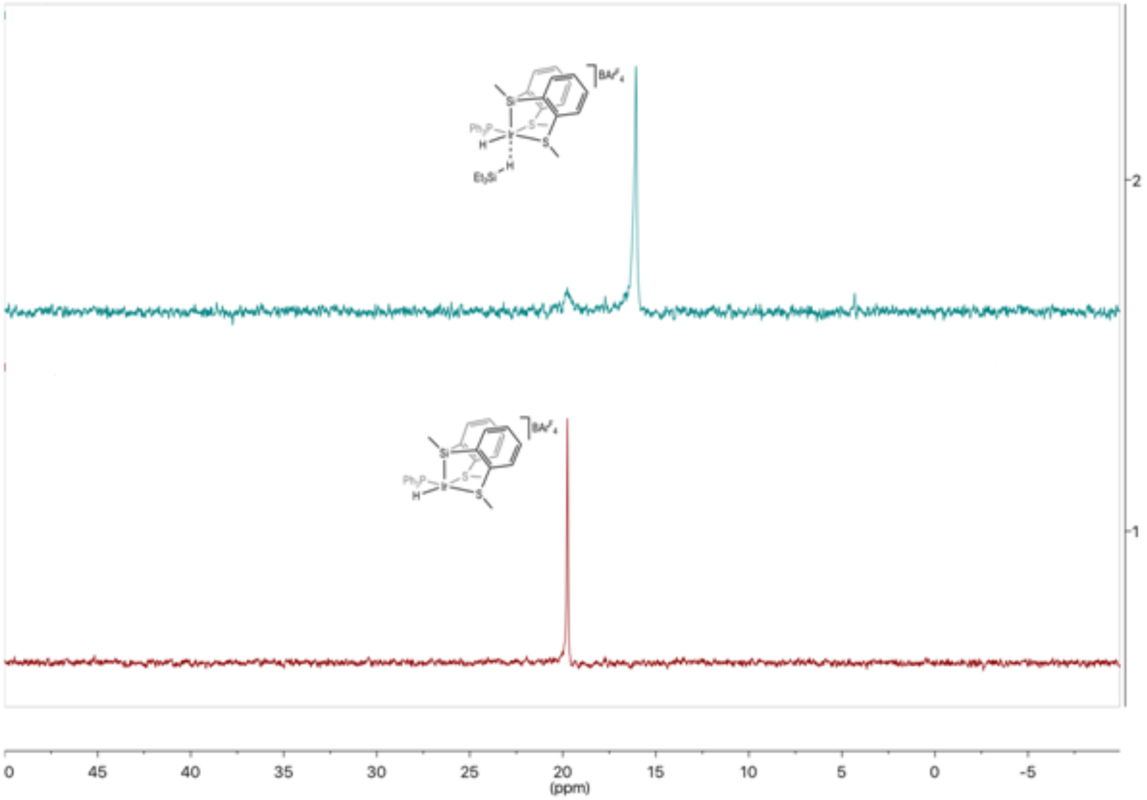

Figure S24. $^{31}P\{^{1}H\}$ NMR spectra of **1** (10 mg, $5.95\cdot10^{-3}$ mmol) dissolved in 0.5 mL of CD_2Cl_2 before (bottom) and after adding 10 eq. of Et_3SiH, compound **3** (top) at -70 ºC.

7. Table of rate constants and TOF½

Table S1. Rate constants and TOF½ obtained for the hydrolysis and alcoholysis of hydrosilanes with iridium catalysts at different temperatures.

Silane	H_2O/Alcohol	Cat	Temp (ºC)	*k* (*s*$^{-1}$*M*$^{-1}$)	*TOF*½ (*h*$^{-1}$)
Et_3SiH	H_2O	[Ir(cod)Cl]$_2$	25	19.06 ± 0.52	6276
Et_3SiH	H_2O	**2**	25	-	
Et_3SiH	H_2O	**1**	25	34.98 ± 0.45	20385
Me_2PhSiH	H_2O	**1**	25	219.23 ± 24.11	135678
$MePh_2SiH$	H_2O	**1**	25	64.23 ± 2.73	44776
Ph_3SiH	H_2O	**1**	25	1.58 ± 0.01	950
Et_3SiH	MeOH	**1**	25	22.31 ± 0.10	13107
Et_3SiH	EtOH	**1**	25	21.63 ± 0.12	11952
Et_3SiH	^{i}PrOH	**1**	25	2.78 ± 0.01	1301
Et_3SiH	H_2O	**1**	30	47.49 ± 1.18	22388
Et_3SiH	H_2O	**1**	20	30.38 ± 0.37	16216
Et_3SiH	H_2O	**1**	15	26.67 ± 0.32	13825
Et_3SiH	H_2O	**1**	10	21.02 ± 0.23	10526
Et_3SiH	H_2O	**1**	5	15.48 ± 0.13	7463
Et_3SiH	H_2O	**1 (0.5 mol %)**	25	8.92 ± 0.22	10526
Et_3SiH	H_2O	**1 (0.1 mol %)**	25	1.38 ± 0.01	8221
Et_3SiH	D_2O	**1**	25	35.19 ± 0.93	17964
Et_3SiD	H_2O	**1**	30	(15.85 ± 0.04) ·10^{-2}	85
Et_3SiD	H_2O	**1**	25	(10.10 ± 0.02) ·10^{-2}	48
Et_3SiD	H_2O	**1**	20	(6.28 ± 0.04) ·10^{-2}	43
Et_3SiD	H_2O	**1**	15	(4.61 ± 0.06) ·10^{-2}	18
Et_3SiD	H_2O	**1**	10	(2.56 ± 0.02) ·10^{-2}	15
Et_3SiD	H_2O	**1**	5	(1.64± 0.04) ·10^{-2}	8
Et_3SiH	D_2O	[Ir(cod)Cl]$_2$	25	17.34 ± 0.47	5268
Et_3SiD	H_2O	[Ir(cod)Cl]$_2$	25	8.45 ± 0.01	2692
Et_3SiH	MeOD	**1**	25	21.20 ± 0.09	11952
Et_3SiD	MeOH	**1**	25	(15.65 ± 0.02) ·10^{-2}	76
Me_2PhSiD	H_2O	**1**	25	50.65 ± 1.5	25714
Ph_3SiD	H_2O	**1**	25	(9.40 ± 0.01) ·10^{-2}	47

8. Theoretical Procedures

All calculations were carried out within the Density Functional Theory (DFT),[4, 5] using the Gaussian16 program package.[6] In order to determine the reaction mechanism, the following procedure was followed. First, geometry optimizations were performed by using the M06 exchange-correlation functional,[7] combined with the 6-31+G(d,p) basis set for the non-metal atoms,[8, 9] and the ECP60MDF Sttutgart-Cologne relativistic core potentials along with the aug-cc-pVDZ-PP basis set for Ir,[10] taking into account solvent effect (THF) by means of the integral equation formalism of the polarized continuum model (IEFPCM).[11] After the geometry optimizations, harmonic vibrational frequencies were obtained by analytical differentiation of gradients, at the same level of theory, to identify whether the characterized structures were true minima. Such frequencies were then used to evaluate the zero-point vibrational energy (ZPVE) and the thermal (T = 298 K) vibrational corrections to the Gibbs free energy (G^{corr}). Then, single-point calculations using the 6-311++G(2df,2p) basis set[12] for non-metal atoms and same ECP combined with aug-cc-pVTZ-PP basis set for Ir,[10] were performed on the optimized structures to refine the electronic energy (E_{elec}). In this vein, the Gibbs free energies (G^{sol}) of each species in solution were calculated as follows:

$$\boxed{G^{sol} = E_{elec} + G^{corr}} \qquad \text{equation (1)}$$

Finally, these free energy values were used to calculate the ΔG values of the reaction mechanism. This reaction mechanism is depicted in Figure 7 of the manuscript, and the corresponding species are depicted in Figure S25.

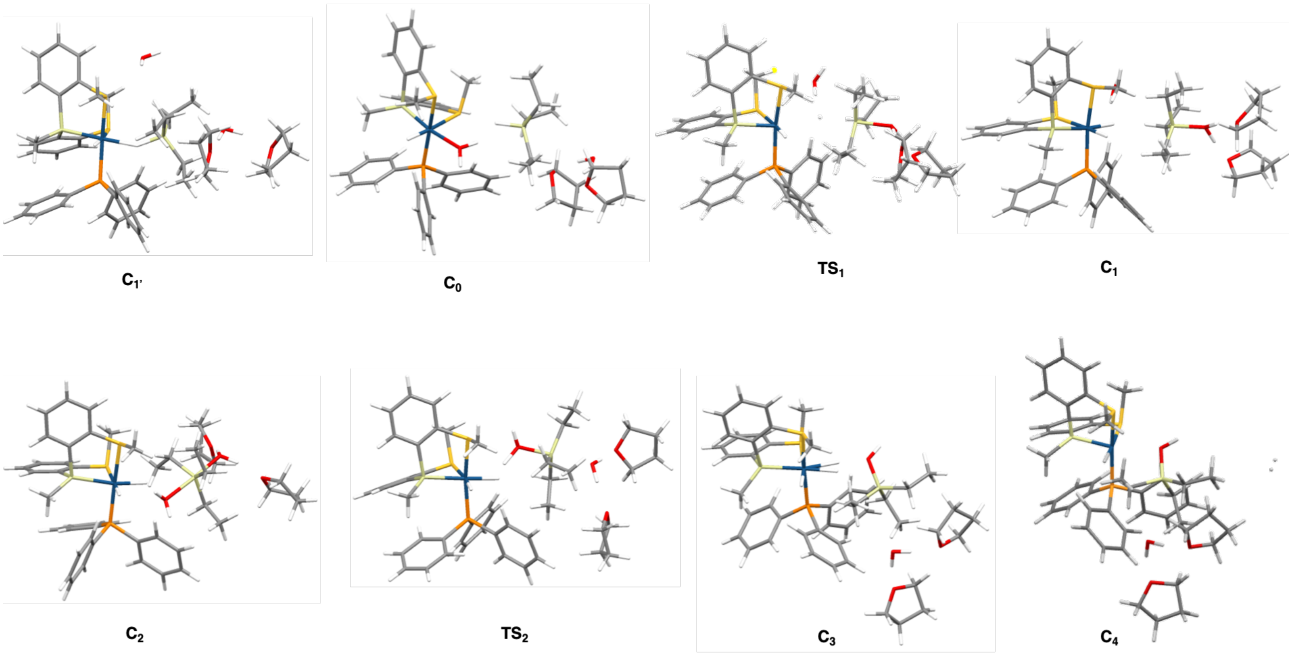

Figure S25. Representation of the calculated geometries corresponding to the intermediates and transition states involved in the mechanism represented in Figure 7 of the manuscript.

9. Calculation of the theoretical Kinetic Isotope Effect by means of Eyring and Bigeleisen-Mayer approaches, including Wigner and Bell Inverse Parabola tunneling corrections

The theoretical Kinetic Isotope Effect (KIE), without the consideration of tunneling effects, is calculated as the ratio between the calculated reaction rate constant for H and D:

$$\mathrm{KIE}_{\mathrm{theo}}^{\mathrm{No\ Tunnel}} = \frac{k_H}{k_D}$$

In order to calculate these reaction rate constants, Transition State Theory is applied. In this vein,

$$k = \frac{k_b\, T Q^{TS}(T)}{h Q_r(T)} e^{-V^{TS}/RT}$$

T is the temperature, k_B stands for the Boltzmann constant, V^{TS} is the potential energy at the saddle point, and finally $Q_r(T)$ and $Q^{TS}(T)$ are harmonic (quantum) partition functions for the reactants and the saddle point. This equation can be rewritten in the following way as function of the free energy change (Eyring equation)

$$k = \frac{k_b\, T}{h} e^{-\Delta G/RT}$$

The calculated energy barriers for H and D are calculated by carrying out frequency calculations for both isotopes on the reactant complexes (C_0) and transition states (TS_1):

$$k_H = \frac{k_b\, T}{h} e^{-\Delta G_H/RT} = 2188.62358\ s^{-1}$$

$$k_D = \frac{k_b\, T}{h} e^{-\Delta G_D/RT} = 636.521429\ s^{-1}$$

Substituting these expressions in the $\mathrm{KIE}_{\mathrm{theo}}^{\mathrm{Eyring}}$ expression we obtain the following:

$$\mathrm{KIE}_{\mathrm{theo}}^{\mathrm{Eyring}} = \frac{k_H}{k_D} = 3.4384$$

Other method to calculate Kinetic Isotope Effects is the Bigeleisen-Mayer method.[13, 14] This method is more sophisticated than the Eyring one, but no tunneling corrections are included. Hence, Wigner tunneling correction[15-17] and the Bell Inverse Parabola[18] correction have been considered as well. Both Wigner and Bell's methods are one dimensional approaches for tunneling.

In Wigner approach, tunnel corrections are considered very simply as a function of the imaginary frequency of the reaction coordinate at the TS structure.

$$k^W = 1 + \left(\frac{h[w]}{k_B T}\right)^2 \frac{1}{24}$$

According to the calculated imaginary frequency for H (-589 cm^{-1}), and D (-428 cm^{-1}) in TS_1, the correction factor would take the following values for k_H^W and k_D^W.

$$k_H^W = 1 + \frac{1}{24}\left(\frac{6.626 * 10^{-34}[589 * 3 * 10^{10}]}{1.30\ 10^{-23}\ 298}\right)^2 = 1.392$$

$$k_D^W = 1 + \frac{1}{24}\left(\frac{6.626 * 10^{-34}[428 * 3 * 10^{10}]}{1.30\ 10^{-23}\ 298}\right)^2 = 1.201$$

Hence, considering Wigner tunneling correction and the calculated imaginary frequencies for H and D, the corrected KIE would be increased by a factor of $\frac{k_H^W}{k_D^W} = 1.159$, which is not enough to explain the large observed experimental KIE values.

In the Bell's approach, the shape of the PES at the TS is approached as an inverse parabola. It is a bit more sophisticated than Wigner's approach, but large errors are associated to these methods for H transfer. Nevertheless, they may be used as indicative of tunneling when they increase the calculated Bigeleisen KIE value.[19] We have calculated the KIE values according to these 3 methods, namely, Bigeleisen-Mayer, Wigner and Bell as implemented in the p-quiver program.[20] and are collected in Table S2 along with the Eyring KIE value.

Table S2: The calculated KIE values using the Eyring, Bigeleisen-Mayer, Wigner and Bell Inverse Parabola methods.

	Eyring	Bigeleisen-Mayer	Wigner	Bell Inverse Parabola
KIE	3.4384	3.3243	3.6449	3.7741

As can be seen, the very approximate tunneling corrections of Wigner and Bell Inverse Parabola slightly increase the KIE, suggesting the importance of tunneling in this reaction. Discussion of the results are included in the manuscript as well.

10. Calculation of the KIE considering quantum tunneling by means of the WBK method

In order to calculate the Potential Energy Surfaces for H/D transfer tunneling, we focused on the imaginary frequency of the TS1 structure. Its value is calculated to be -589 cm^{-1} for H transfer. Visualization of this normal mode shows the H transfer from $HSi(Et)_3$ to Ir catalyst, as expected for this H/D transfer process. IRC calculations[21,22] confirmed this fact. Hence, in this process, the water molecule coordinated to the catalyst in C_0 must be decoordinated while H is transferred. We have calculated the PES for such process by means of geometry optimizations with restrictions, without considering any further correction in the electronic energies. First, the Ir-Si distance was reduced by 0.1 A, until the water decomplexation was observed. This occurred at Ir-Si distance of 4.4 A approximately, similar to the Ir-Si distance in the optimized TS_1 structure. Then, Ir-H distance was reduced systematically, by 0.1 A, fixing the Ir-Si distance to 4.4 A. Calculated PES is depicted in Figure S26. Notice that the maximum of the curve (ΔE of around 55 kJ/mol) is in agreement with the calculated barrier for TS_1 (ΔG = 53.93 kJ/mol).

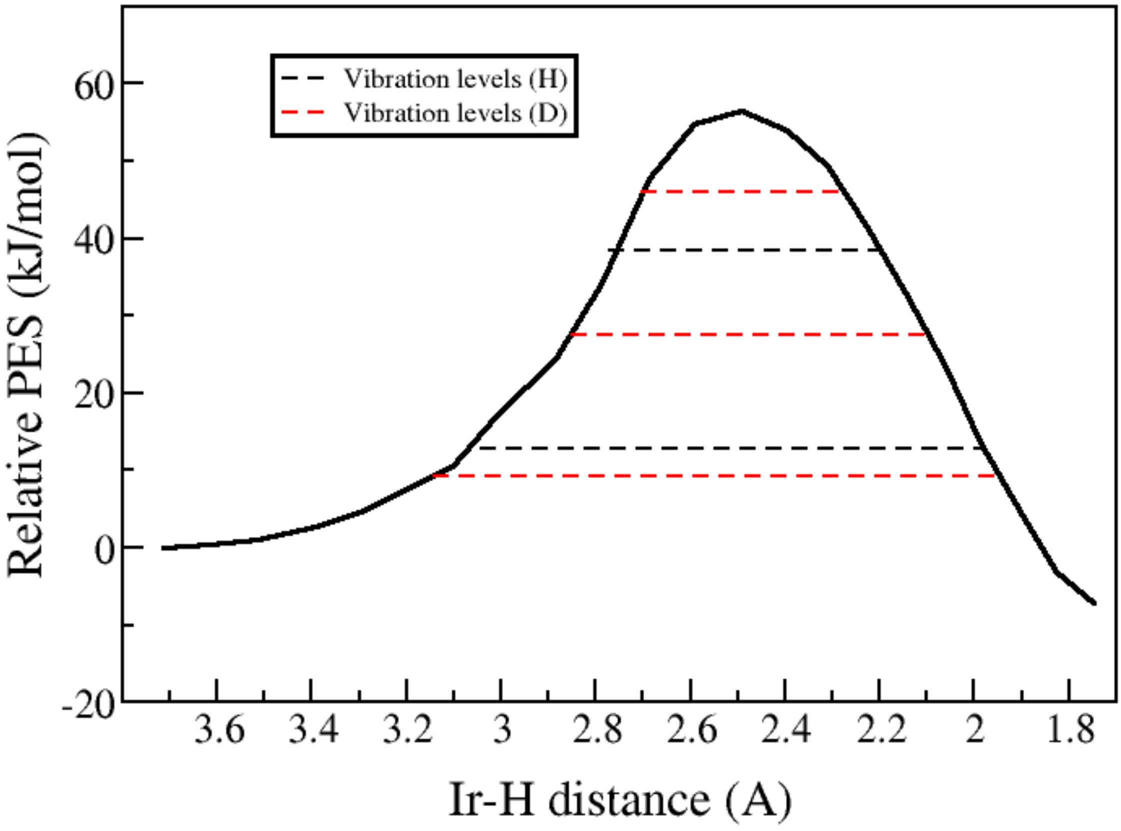

Figure S26: Schematic representation of calculated PES for H/D tunneling from C_0 to C_1. Black dashed lines stand for H vibrational levels, and red dashed lines for D vibrational levels.

10.1 Tunneling at 298 K

Hence, we have used this PES for calculating the tunneling effects via the Wentzel-Kramers-Brillouin WBK semiclassical approach.[23] First of all, we calculate all the available vibrational levels for H and D, from which tunneling may occur. These vibrational levels are those corresponding to the Si-H(D) stretching in the H(D)Si(Et)$_3$ moiety in C$_0$, as calculated by Gaussian 16. According to the harmonic approximation, the vibrational energy levels are calculated to be:

$$G(v) = \left(v + \frac{1}{2}\right)\bar{v}_e$$

Harmonic vibrational frequencies for the mentioned vibrational modes are calculated to be 2146.2 cm^{-1} (25.76 kJ/mol) and 1539.3 cm^{-1} (18.4 kJ/mol) for H and D, respectively. Representing these energy levels in the calculated PES, two vibrational levels are available for H (at 12.84 kJ/mol and 38.51 kJ/mol, black dashed lines in Figure S26) and three for D (at 9.21 kJ/mol, 27.62 kJ/mol and 46.04 kJ/mol, red dashed lines in Figure S26). We are aware that this harmonic approach is accurate near C$_0$, while the accuracy decreases near TS1 due to the anharmonicity of the PES. Nevertheless, from a qualitative point of view this approach is sufficient to take into account the tunneling effects from excited vibrational levels. Hence, in order to calculate the tunneling transmission coefficient for H and D, the probability of tunneling from all available energy levels should be considered, along with the population of each level.

According to the Wentzel-Kramers-Brillouin (WKB) semiclassical approach,[23] the probability of tunneling from each level can be calculated by the next expression

$$P(E) = e^{-2\int_{x_1}^{x_2} dx \sqrt{\frac{2m}{\hbar^2}(V(x)-E)}}$$

being V(x) the calculated one-dimensional PES and E the energy associated to the vibrational level. The population of each vibrational level (PB$_i$) can be calculated according to Boltzmann distribution function for a given temperature. The calculated values are collected in Table S3.

Table S3: Population of different vibrational levels at 298 K, and the calculated probability of tunneling from each level, for H and D.

H			D		
Vib. Level (v)	PB$_v$	P$_v$(E)	Vib. Level (v)	PB$_v$	P$_v$(E)
0	0.999968	1.35922 x 10^{-10}	0	0.999405	2.48568 x 10-^{16}
1	3.18287 x 10^{-5}	2.54155 x 10^{-4}	1	5.94487 x 10^{-4}	3.21735 x 10^{-9}
-	-	-	2	3.53625 x 10^{-7}	1.20253 x 10^{-3}

Having into account these values, the transmission probability (τ) is calculated by averaging the tunneling probability from each energy level taking into account its population.

$$\tau = \sum_{v=0}^{n} PB_v \cdot P_v(E)$$

Notice that in this formulation the value of τ ranges from 0 to 1, and its physical meaning is to provide the probability of tunneling per event. Hence, in order to obtain the effect of tunneling in the rate constant, one should multiply this probability by the number of events occurring during a second, i.e. the vibrational

frequency. So, the tunneling rate (k^{tun}) would be obtained as the product of □ and the frequency the vibration takes place:

$$k^{tun} = \bar{\nu_e} \cdot \tau$$

In this vein, in order to move from C0 to C1 in the reaction mechanism, H/D have two paths. One, moving above the barrier (Eyring or Bigeleisen), and the other by moving through tunneling. Hence, the reaction rate constant would be the sum of both events.

$$k = k^{Eyr} + k^{tun}$$

If the tunneling effect is negligible, then the rate constant is governed by the barrier height, but as tunneling importance increases its influence in the rate constant is more important. Taking this into account, we may define the total theoretical Kinetic Isotopic Effect as

$$KIE^{Theo} = \frac{k_H}{k_D} = \frac{k_H^{Ey} + k_H^{tun}}{k_D^{Ey} + k_D^{tun}}$$

It could be compared it to KIE without considering tunneling, regardless considering Eyring or Bigeleisen methods (KIE^{Ey}, KIE^{B}) and the KIE considering only tunneling:

$$KIE^{Ey} = \frac{k_H^{Ey}}{k_D^{Ey}}$$

$$KIE^{tun} = \frac{k_H^{tun}}{k_D^{tun}}$$

In Table S4 we provide all these results, calculated according to the procedure described above.

Table S4: Calculated transmission probability (τ), tunneling and no-tunneling rate constans (k^{tun} and k^{Eyr}) in s^{-1}, and calculated Eyring KIE (KIE^{Eyr}), Bigeleisen KIE (KIE^{B}), tunneling KIE (KIE^{tun}) and total theoretical KIE (KIE^{theo}).

	τ	k^{tun} (s^{-1})	k^{Eyr} (s^{-1})	KIE^{Eyr} (KIE^{B})	KIE^{tun}	KIE^{theo}	KIE^{exp}
H	8.22534×10^{-9}	3.32199×10^{6}	2.2117×10^{3}	3.44 (3.32)	26.85	26.73	346
D	4.27159×10^{-10}	1.23739×10^{5}	6.43631×10^{2}				

According to the calculated values, we observe that $k^{tun} \gg k^{Ey}$, and, as a consequence, $KIE^{theo} \approx KIE^{tun}$. Hence, tunnelling is crucial to understand the large experimental KIE values.

10.2 Influence of the Temperature in the KIE

The experimental KIEs are affected by the Temperature, and we have calculated also the theoretical KIE^{theo} at different temperatures (assuming that $KIE^{theo} \approx KIE^{tun}$),, and compared to the experimental ones (KIE^{Exp}). First, we collect the occupation of vibrational levels and their corresponding tunneling probability for different temperatures, in Table S5, and then, in Table S6, the calculated transmission probability, tunneling rate constans (k^{tun}) in s^{-1}, and calculated Eyring KIE (KIE^{Eyr}), tunneling KIE (KIE^{tun}) and total theoretical KIE (KIE^{theo}), at different temperatures (K) are given.

Table S5: Population of different vibrational levels at 278, 283, 288, 293, 298 and 303 K, and the calculated probability of tunneling from each level, for H and D.

	H			D		
T	Vib. Level (v)	PB_v	$P_v(E)$	Vib. Level (v)	PB_v	$P_v(E)$
278	0	0.999985	1.35922×10^{-10}	0	0.999651	2.48568×10^{-16}
	1	1.51169×10^{-5}	2.54155×10^{-4}	1	3.48592×10^{-4}	3.21735×10^{-9}
	-	-	-	2	1.21559×10^{-7}	1.20253×10^{-3}
283	0	0.999982	1.35922×10^{-10}	0	0.999599	2.48568×10^{-16}
	1	1.83901×10^{-5}	2.54155×10^{-4}	1	4.01189×10^{-4}	3.21735×10^{-9}
	-	-	-	2	1.61017×10^{-7}	1.20253×10^{-3}
288	0	0.999978	1.35922×10^{-10}	0	0.99954	2.48568×10^{-16}
	1	2.22204×10^{-5}	2.54155×10^{-4}	1	4.59473×10^{-4}	3.21735×10^{-9}
	-	-	-	2	2.11212×10^{-7}	1.20253×10^{-3}
293	0	0.999973	1.35922×10^{-10}	0	0.999476	2.48568×10^{-16}
	1	2.66758×10^{-5}	2.54155×10^{-4}	1	5.2379×10^{-4}	3.21735×10^{-9}
	-	-	-	2	2.745×10^{-7}	1.20253×10^{-3}
298	0	0.999968	1.35922×10^{-10}	0	0.999405	2.48568×10^{-16}
	1	3.18287×10^{-5}	2.54155×10^{-4}	1	5.94487×10^{-4}	3.21735×10^{-9}
	-	-	-	2	3.53625×10^{-7}	1.20253×10^{-3}
303	0	0.999962	1.35922×10^{-10}	0	0.999328	2.48568×10^{-16}
	1	3.77564×10^{-5}	2.54155×10^{-4}	1	6.71909×10^{-4}	3.21735×10^{-9}
	-	-	-	2	4.51765×10^{-7}	1.20253×10^{-3}

Table S6: Calculated transmission probability (τ), tunneling rate constans (k^{tun}) in s^{-1}, and calculated tunneling KIE (KIE^{tun}) and total theoretical KIE (KIE^{theo}), different temperatures (K).

T		τ	k^{tun} (s^{-1})	KIE^{tun}	KIE^{Exp}
278	H	3.9780 x 10^{-9}	1.6066 x 10^{6}	37.65	943
	D	1.4730 x 10^{-10}	4.2670 x 10^{4}		
283	H	4.8099 x 10^{-9}	1.9426 x 10^{6}	34.40	821
	D	1.9492 x 10^{-10}	5.6464 x 10^{4}		
288	H	5.7834 10^{-9}	2.3357 x10^{6}	31.56	578
	D	2.5547 10^{-10}	7.4004 10^{4}		
293	H	6.9157 x 10^{-9}	2.7931 x 10^{6}	29.06	487
	D	3.3178 x 10^{-10}	9.6110 x 10^{4}		
298	H	8.2253 x 10^{-9}	3.3212 x 10^{6}	26.85	346
	D	4.2716 x 10^{-10}	1.2374 $10x^{5}$		
303	H	9.7319 x 10^{-9}	3.9304 x 10^{6}	24.88	299
	D	5.4542 x 10^{-10}	1.5800 x 10^{5}		

As can be observed from the values given in Table S6, the behavior of the KIE^{exp} with temperature is correctly described by the KIE^{theo}. This behavior comes from the fact that k_D^{tun} increases with the temperature more than k_H^{tun}, once higher vibrational levels become available for D. As a consequence, the KIE decreases.

11. References

[1] a) Synthesis of {Ir[SiMe(o-C_6H_4SMe)$_2$](H)(PPh_3)(THF)}[BAr$^F{}_4$] (**1**) and {IrCl[SiMe(o-C_6H_4SMe)$_2$](H)(PPh_3)} (**2**): S. Azpeitia, A. Rodriguex-Dieguez, M. A. Garralda, M. A. Huertos, *ChemCatChem,* **2018**, *10*, 2210. b) Synthesis of Me_2PhSiD and Ph_3SiD: N. Gandhamsetty, S. Park, S. Chang, *J. Am. Chem. Soc.* **2015**, *137*, 15176.

[2] www.manonthemoontech.com

[3] L. Falvine, R. Credendino, A. Poater, A. Petta, L. Serra, R. Oliva, V.Scarano, L. Cavallo. *Organometallics*, **2016**, *35*, 2286.

[4] P. Hohenberg, W. Kohn, *Physical Review*, **1964**, *136 (3B),* B864-B871.

[5] W. Kohn, L. J. Sham, *Physical Review* **1965**, *140 (4A)*, A1133-A1138.

[6] M. J. Frisch, G. W. Trucks, H. B. Schlegel, G. E. Scuseria, M. A. Robb, J. R. Cheeseman, G. Scalmani, V. Barone, G. A. Petersson, H. Nakatsuji, X. Li, M. Caricato, A. V. Marenich, J. Bloino, B. G. Janesko, R. Gomperts, B. Mennucci, H. P. Hratchian, J. V. Ortiz, A. F. Izmaylov, J. L. Sonnenberg, F. Williams, F. Ding, F. Lipparini, F. Egidi, J. Goings, B. Peng, A. Petrone, T. Henderson, D. Ranasinghe, V. G. Zakrzewski, J. Gao, N. Rega, G. Zheng, W. Liang, M. Hada, M. Ehara, K. Toyota, R. Fukuda, J. Hasegawa, M. Ishida, T. Nakajima, Y. Honda, O. Kitao, H. Nakai, T. Vreven, K. Throssell, J. A. Montgomery Jr., J. E. Peralta, F. Ogliaro, M. J. Bearpark, J. J. Heyd, E. N. Brothers, K. N. Kudin, V. N. Staroverov, T. A. Keith, R. Kobayashi, J. Normand, K. Raghavachari, A. P. Rendell, J. C. Burant, S. S. Iyengar, J. Tomasi, M. Cossi, J. M. Millam, M. Klene, C. Adamo, R. Cammi, J. W. Ochterski, R. L. Martin, K. Morokuma, O. Farkas, J. B. Foresman, D. J. Fox, Gaussian 16 Rev. B.01, Wallingford, CT, **2016**.

[7] Y. Zhao, D. G. Truhlar, *Theor. Chem. Acc.*, **2008**, *120*, 215.

[8] P. C. Hariharan, J. A. Pople, *Theor. Chem. Acc.*, **1973,** *28*, 213.

[9] M. M. Francl, W. J. Pietro, W. J. Hehre, J. S. Binkley, D. J. DeFrees, J. A. Pople, M. S. Gordon, *J. Chem. Phys.,* **1982**, *77*, 3654.

[10] D. Figgen, K. A. Peterson, M. Dolg, H. Stoll, *J. Chem. Phys*. **2009**, *130*, 164108.

[11] G. Scalmani, M. J. Frisch, *J. Chem. Phys.* **2010**, *132*, 114110.

[12] R. Krishnan, J. S. Binkley, R. Seeger, J. A. Pople, *J. Chem. Phys*. **1980**, *72*, 650.

[13] J. Bigeleisen and M. G. Mayer, *J. Chem. Phys.* **1947**, *15,* 261.

[14] M. Wolfsberg, *Acc. Chem. Res*. **1972**, *5*, 225.

[15] E. P. Wigner, Z. *Phys. Chem.*, Abt. 6, *19*, 203 (**1932**).

[16] B. C. Garrett and D. G. Truhlar, *J. of Phys. Chem.,* 83, *200*, **1979**

[17] R. P. Bell, The tunnel effect in Chemistry, ISBN 978-0-412-21340-3, Chapman and Hall, **1980**.

[18] R. P. Bell. *Chem. Soc. Rev.* **1974**, *3*, 513.

[19] J.E. Bercaw, G. S. Chen, J. A. Labinger and B. -L. Lin, *Organometallics*, **2010**, *29*, 4354-4359.

[20] T. L. Anderson, E. E. Kwan, PyQuiver **2020**, www.github.com/ekwan/PyQuiver

[21] K. Fukui, *Acc. Chem. Res*. **1981**, *14*, 363-368.

[22] H. P. Hratchian and H. B. Schlegel, in Theory and Applications of Computational Chemistry: The First 40 Years, Ed. C. E. Dykstra, G. Frenking, K. S. Kim, and G. Scuseria (Elsevier, Amsterdam, **2005**) 195-249.

[23] N. Froman and P. O. Froman, Wkb approximation, contributions to the theory; North Holland Publishing Company: Amsterdam, **1965**.

12. Cartesian Coordinates

2-THF at Solv/Set2

- Compound: **C'$_1$**

Symbol	X	Y	Z
C	-6.189285	-1.139725	-1.946509
O	-6.042703	0.082100	-1.210854
C	-6.450583	1.196272	-2.018295
C	-6.597322	0.646735	-3.425944
C	-7.043747	-0.786110	-3.149831
O	-5.445295	1.342127	1.290704
Si	-1.843401	0.754433	0.885862
C	-1.302746	1.821986	2.338290
C	-2.441889	2.689957	2.878827
C	-2.825807	-0.712160	1.537596
C	-3.535889	-1.578689	0.503831
C	-2.649713	1.779700	-0.471556
C	-2.840347	1.069424	-1.811933
O	-7.880523	0.452861	2.382016
C	-7.930936	-0.842520	1.763581
C	-8.836475	-0.686506	0.554622
C	-9.808652	0.385994	1.033741
C	-8.874620	1.307083	1.796166
O	0.098654	4.929206	1.590580
C	2.575840	2.646308	2.261561
S	2.235844	0.878066	1.933078
C	3.939319	0.296471	1.756754
C	4.549696	0.299456	0.495225
C	5.883429	-0.121205	0.429328
C	6.565057	-0.550094	1.567464
C	5.920372	-0.571892	2.803015
C	4.598074	-0.141486	2.903931
Si	3.503076	0.793073	-1.013861
C	4.224229	0.092547	-2.598465
Ir	1.296190	0.423139	-0.363458
S	0.923382	2.747214	-0.896744
C	2.550586	3.510147	-1.056580
C	3.674467	2.689138	-1.141052
C	4.918448	3.326172	-1.251861
C	5.020528	4.715127	-1.272490
C	3.874077	5.504760	-1.181108
C	2.624437	4.902411	-1.069444
P	1.415138	-1.900497	-0.159996
C	-0.073194	-2.740689	-0.855378
C	-0.534113	-3.935055	-0.287693
C	-1.598563	-4.625659	-0.862226
C	-2.214043	-4.135993	-2.011864
C	-1.763650	-2.947542	-2.582265
C	-0.703372	-2.253144	-2.005652
C	2.791566	-2.755074	-1.023411

C	4.065004	-2.808545	-0.443877
C	5.125484	-3.401969	-1.121766
C	4.929335	-3.943253	-2.390720
C	3.666844	-3.889551	-2.977040
C	2.602425	-3.300804	-2.297741
C	1.470925	-2.551388	1.558511
C	0.685860	-1.902575	2.516718
C	0.625194	-2.364971	3.826989
C	1.358553	-3.489708	4.198668
C	2.132240	-4.154875	3.249767
C	2.183660	-3.695256	1.935677
C	0.407384	2.815192	-2.646624
H	-5.585352	0.811910	0.487647
H	-6.220888	1.137916	1.844462
H	-0.579965	0.155893	0.221215
H	-3.614350	2.136131	-0.080788
H	-2.026207	2.681856	-0.595545
H	-3.150713	1.768421	-2.600761
H	-3.615548	0.295457	-1.740632
H	-1.917248	0.576479	-2.151822
H	-4.315111	-1.005156	-0.011508
H	-2.844059	-1.954863	-0.260864
H	-4.018614	-2.449942	0.966188
H	-2.145098	-1.319398	2.154922
H	-3.563798	-0.274042	2.227508
H	-0.463936	2.465329	2.035976
H	-0.918710	1.161702	3.132470
H	-2.785776	3.410815	2.124838
H	-3.316930	2.091081	3.160517
H	-2.128303	3.260976	3.761424
H	0.883352	0.100681	-1.870693
H	-0.568685	2.327971	-2.720648
H	0.328888	3.865584	-2.939671
H	1.141689	2.290110	-3.263217
H	3.623347	0.444834	-3.446547
H	5.252027	0.450056	-2.747189
H	4.232085	-1.002509	-2.612339
H	1.723012	5.507231	-0.981577
H	3.952912	6.588915	-1.186867
H	5.997569	5.186184	-1.351178
H	5.827917	2.726573	-1.310149
H	3.251842	3.057519	1.505842
H	1.625743	3.192424	2.249212
H	3.030737	2.714975	3.253277
H	4.079359	-0.154662	3.861839
H	6.442824	-0.925898	3.688280
H	7.597245	-0.884008	1.488224
H	6.393002	-0.146173	-0.534672
H	4.238943	-2.387768	0.544902
H	6.107164	-3.436575	-0.653861
H	5.757831	-4.406846	-2.920599
H	3.504668	-4.310893	-3.966129
H	1.621295	-3.274690	-2.768176
H	0.112360	-1.022709	2.236030

H	0.007055	-1.842246	4.553936
H	1.322207	-3.851484	5.223335
H	2.695361	-5.042089	3.529026
H	2.774079	-4.244296	1.205749
H	-0.362920	-1.323271	-2.457030
H	-2.242047	-2.550837	-3.475308
H	-3.046033	-4.675718	-2.458128
H	-1.946989	-5.549078	-0.405895
H	-0.066638	-4.336143	0.609544
H	-0.623606	5.282583	2.121763
H	-0.314970	4.605261	0.780396
H	-8.335136	-1.564284	2.488272
H	-6.905512	-1.146304	1.506488
H	-9.317152	-1.625101	0.260932
H	-8.250325	-0.310304	-0.295440
H	-10.331245	0.898629	0.220040
H	-10.559863	-0.045889	1.707866
H	-9.357382	1.874584	2.598823
H	-8.376575	2.017585	1.114791
H	-5.191521	-1.497434	-2.253544
H	-6.634152	-1.892866	-1.282897
H	-6.886368	-1.466125	-3.992240
H	-8.109485	-0.809882	-2.884363
H	-7.305651	1.220215	-4.030919
H	-5.625813	0.649056	-3.938989
H	-5.694786	1.986768	-1.923314
H	-7.407362	1.582931	-1.628614

- Compound: **C_0**

Symbol	X	Y	Z
C	0.751606	-3.523529	1.437762
C	0.542922	-2.448620	0.567288
C	-0.713230	-1.830965	0.547241
C	-1.733624	-2.259187	1.391098
C	-1.506472	-3.318166	2.269040
C	-0.266283	-3.950357	2.287839
P	1.789339	-1.850355	-0.648082
C	1.213823	-2.680057	-2.192633
C	0.596754	-3.935867	-2.114700
C	0.209785	-4.605013	-3.271328
C	0.428950	-4.028132	-4.521481
C	1.041261	-2.781262	-4.609477
C	1.434249	-2.110826	-3.451960
Ir	1.934812	0.452194	-0.923186
S	2.043647	2.759233	-1.588771
C	2.836744	2.768517	-3.230698
S	0.790317	0.959531	1.254119
C	0.784279	2.744711	1.646072
C	2.037051	0.417951	2.448803
C	1.589206	0.020712	3.707158
C	2.520961	-0.390049	4.659282
C	3.879307	-0.386942	4.346354
C	4.305785	0.005262	3.077717
C	3.393398	0.404179	2.093234
Si	3.856149	0.819327	0.293454
C	5.507761	0.066236	-0.187077
C	4.128742	2.709359	0.229933
C	3.345365	3.517047	-0.596789
C	3.491610	4.903492	-0.656064
C	4.466436	5.511375	0.129126
C	5.266054	4.734927	0.968182
C	5.094510	3.353820	1.016516
C	3.327831	-2.756936	-0.239427
C	4.074924	-3.370167	-1.250274
C	5.284792	-3.993045	-0.950371
C	5.757570	-4.012412	0.359804
C	5.020021	-3.399272	1.371363
C	3.815209	-2.770622	1.073403
O	-0.078155	0.520637	-2.316101
O	-4.933361	-1.162242	1.868776
O	-7.003287	0.717149	1.268275
O	-7.301773	0.226662	-1.483090
C	-4.000435	2.073030	2.777057
C	-2.822897	1.481323	2.002312
Si	-2.911223	1.802567	0.140372
C	-3.563556	3.532007	-0.224917
C	-2.639949	4.638804	0.283296
C	-3.896885	0.489895	-0.785645
C	-3.979891	0.729087	-2.292130

H	0.522510	0.023330	3.933012
H	2.184799	-0.713357	5.641364
H	4.607476	-0.704992	5.089192
H	5.370237	-0.029893	2.842217
H	5.723626	2.765960	1.686715
H	6.020749	5.210544	1.590183
H	4.593947	6.590401	0.093931
H	2.848507	5.501404	-1.300198
H	5.737250	0.374798	-1.215387
H	6.313627	0.437753	0.459658
H	5.507835	-1.027786	-0.146705
H	2.178842	2.206044	-3.898684
H	2.928966	3.801995	-3.574884
H	3.814830	2.284752	-3.163111
H	0.127769	3.236903	0.921514
H	0.380521	2.870612	2.655205
H	1.793326	3.163896	1.589021
H	3.716449	-3.366904	-2.278520
H	5.856042	-4.467002	-1.744785
H	6.699827	-4.502548	0.592264
H	5.382844	-3.402466	2.397161
H	3.260874	-2.283396	1.874506
H	1.704453	-4.047003	1.451023
H	-0.088153	-4.786078	2.960210
H	-2.298811	-3.658457	2.932954
H	-2.705306	-1.762417	1.368131
H	-0.902517	-1.006169	-0.140016
H	1.923844	-1.141403	-3.530167
H	1.218363	-2.325196	-5.580499
H	0.122073	-4.550774	-5.424170
H	-0.266367	-5.579514	-3.194695
H	0.416330	-4.399932	-1.146256
H	2.823339	0.089388	-2.201938
H	-1.500562	1.762403	-0.405245
H	-4.909483	0.456093	-0.356650
H	-3.458499	-0.498315	-0.571989
H	-4.505286	-0.084642	-2.806431
H	-4.521422	1.655978	-2.521494
H	-2.988104	0.821464	-2.758173
H	-4.564594	3.620780	0.225257
H	-3.711283	3.632461	-1.309884
H	-3.054847	5.638790	0.108706
H	-2.451102	4.550770	1.362425
H	-1.664357	4.600509	-0.221568
H	-2.778385	0.390650	2.153382
H	-1.875613	1.878890	2.395128
H	-3.956938	1.812876	3.842556
H	-4.014325	3.168830	2.714705
H	-4.964890	1.720435	2.387549
H	-6.255665	0.171240	1.569137
H	-7.076714	0.512547	0.317462
C	-5.514806	-2.291272	1.212928
C	-7.415564	1.571154	-1.965426
H	-0.393752	-0.303468	-2.712119

H	-0.830461	0.919649	-1.849343
C	-8.872227	1.933478	-1.759383
H	-6.706582	2.190968	-1.400071
H	-7.138423	1.605428	-3.031596
H	-9.194449	2.778771	-2.374822
C	-9.553176	0.617646	-2.125611
H	-9.052092	2.183069	-0.704650
H	-10.560896	0.516673	-1.712122
H	-9.623885	0.521541	-3.216566
C	-8.587839	-0.416881	-1.558005
H	-8.500180	-1.316068	-2.179282
H	-8.868920	-0.725454	-0.539517
H	-5.861696	-1.959345	0.227086
H	-4.737194	-3.060213	1.066136
C	-6.629950	-2.792436	2.139072
H	-6.612327	-3.884031	2.218685
C	-6.331713	-2.104224	3.485800
H	-7.617110	-2.505290	1.761774
H	-7.073650	-1.324550	3.692215
H	-6.332169	-2.798500	4.331516
C	-4.965008	-1.471584	3.260906
H	-4.148324	-2.176293	3.497783
H	-4.794705	-0.542276	3.815112

- Compound: **TS_1**

Symbol	X	Y	Z
C	-2.944616	3.500785	-0.163706
C	-3.854756	2.658647	0.475942
C	-5.124205	3.188980	0.749109
C	-5.455967	4.496106	0.402320
C	-4.521791	5.309103	-0.240155
C	-3.256392	4.809892	-0.532915
Si	-3.403115	0.841844	0.871982
C	-4.646365	-0.151947	-0.162447
C	-4.318157	-0.463561	-1.487127
C	-5.209158	-1.117072	-2.337481
C	-6.467729	-1.476004	-1.860650
C	-6.813520	-1.198729	-0.539265
C	-5.910232	-0.544482	0.295987
S	-2.670587	-0.078771	-2.132723
C	-3.075886	1.402377	-3.121035
S	-1.292998	2.881424	-0.548054
C	-0.391090	3.649170	0.841216
Ir	-1.326907	0.523362	-0.083380
P	-1.050176	-1.682570	0.599167
C	-0.224968	-2.792384	-0.614650
C	0.495840	-2.252709	-1.681468
C	1.152521	-3.089274	-2.583441
C	1.092738	-4.470608	-2.425845
C	0.375451	-5.018073	-1.361667
C	-0.281784	-4.185284	-0.462835
C	-2.546550	-2.673302	1.012823
C	-2.936138	-2.936520	2.327880
C	-4.101457	-3.657975	2.588079
C	-4.879354	-4.134268	1.537290
C	-4.488723	-3.889039	0.221199
C	-3.334914	-3.159749	-0.038525
C	-0.004912	-1.825531	2.111575
C	1.078597	-2.705233	2.187500
C	1.837049	-2.794284	3.354803
C	1.510973	-2.020152	4.464422
C	0.431636	-1.139464	4.398156
C	-0.309482	-1.032921	3.226375
C	-3.739990	0.605693	2.708107
O	0.142000	1.372977	-3.691912
C	3.222919	0.090944	-2.712054
C	3.864913	0.910530	-3.830386
Si	2.967670	1.013086	-1.083214
O	4.961951	1.267884	-0.921190
C	2.760125	2.896470	-1.143906
C	3.365433	3.717188	-0.006401
C	2.908538	-0.065063	0.464896
C	2.629872	0.675338	1.769693
O	5.951324	1.573132	1.592858
O	6.248715	-0.959838	-1.611232

C	6.082132	2.800989	2.333773
H	5.299807	1.438810	-0.006492
C	6.751264	-1.330051	-2.908066
H	5.461781	0.477910	-1.258814
H	0.932782	0.815019	-1.205180
H	3.228830	3.198832	-2.093708
H	1.692071	3.125572	-1.250341
H	3.172509	4.787934	-0.146452
H	4.453752	3.592962	0.049797
H	2.953299	3.438758	0.972640
H	3.417913	1.407725	1.993266
H	1.670538	1.210394	1.724238
H	2.569685	-0.017692	2.620402
H	2.136492	-0.827988	0.294343
H	3.865447	-0.610201	0.527562
H	2.256973	-0.298885	-3.053260
H	3.842757	-0.788717	-2.470547
H	3.212726	1.735828	-4.143382
H	4.822097	1.353543	-3.527268
H	4.053316	0.295438	-4.718826
H	-0.579731	0.884510	1.273579
H	0.632093	3.264734	0.813155
H	-0.389976	4.734042	0.702744
H	-0.872405	3.372195	1.783355
H	-3.029278	1.207115	3.289947
H	-4.749833	0.953565	2.963631
H	-3.655475	-0.442251	3.020048
H	-2.521568	5.428844	-1.046036
H	-4.780848	6.327043	-0.520715
H	-6.448522	4.881402	0.624323
H	-5.875452	2.564867	1.234706
H	-3.557893	2.159442	-2.493902
H	-2.126034	1.764544	-3.526158
H	-3.744699	1.103786	-3.932607
H	-4.916355	-1.357515	-3.358905
H	-7.168567	-1.988054	-2.515796
H	-7.787786	-1.497286	-0.158300
H	-6.195854	-0.343893	1.329351
H	-3.040163	-2.985091	-1.073168
H	-5.086142	-4.262101	-0.607727
H	-5.784531	-4.701545	1.741006
H	-4.391142	-3.855354	3.617597
H	-2.329053	-2.592023	3.163238
H	0.547207	-1.168403	-1.796819
H	1.706843	-2.657257	-3.414252
H	1.601045	-5.122994	-3.132071
H	0.325462	-6.096677	-1.232657
H	-0.839498	-4.624336	0.363614
H	-1.130578	-0.318870	3.178246
H	0.172507	-0.524450	5.256758
H	2.098743	-2.096452	5.376254
H	2.683794	-3.475960	3.392295
H	1.351516	-3.319867	1.332227
H	0.386683	1.200582	-2.758132

H	0.563448	2.210611	-3.912668
C	7.887771	-2.291415	-2.624546
H	5.923381	-1.763171	-3.480218
H	7.106829	-0.427348	-3.427543
H	8.604117	-2.348292	-3.449189
C	8.476396	-1.696847	-1.348668
H	7.497280	-3.299593	-2.435990
H	9.081208	-2.404266	-0.774246
H	9.102770	-0.828447	-1.589164
C	7.228747	-1.261347	-0.598873
H	7.379250	-0.364096	0.017323
H	6.827120	-2.061409	0.039352
C	6.615163	2.393653	3.694035
H	6.741342	3.472449	1.773211
H	5.089126	3.270413	2.417449
H	6.355073	3.112888	4.475910
C	5.964242	1.026387	3.881063
H	7.707966	2.296150	3.665939
H	6.462456	0.402080	4.628353
H	4.911831	1.139689	4.173983
C	6.068816	0.447828	2.483040
H	5.280564	-0.275508	2.235371
H	7.047101	-0.029444	2.320564

- Compound: **C1**

Symbol	X	Y	Z
C	-2.774740	3.688138	0.063096
C	-3.731576	2.947665	0.757404
C	-4.862926	3.651739	1.200126
C	-5.024877	5.012277	0.953195
C	-4.052622	5.714772	0.239758
C	-2.919604	5.048963	-0.215708
Si	-3.509971	1.046822	0.991048
C	-4.903873	0.361811	-0.134265
C	-4.605454	0.004594	-1.452672
C	-5.573958	-0.471863	-2.337125
C	-6.891747	-0.596069	-1.905478
C	-7.221965	-0.258190	-0.593687
C	-6.237683	0.212916	0.272739
S	-2.893700	0.071408	-2.061159
C	-3.021796	1.546508	-3.128743
S	-1.261420	2.874390	-0.515782
C	-0.123147	3.529203	0.752144
Ir	-1.441532	0.521306	-0.080457
P	-1.358067	-1.690985	0.517910
C	-0.683548	-2.896912	-0.710170
C	0.016963	-2.461293	-1.835255
C	0.535521	-3.378150	-2.749629
C	0.358550	-4.743132	-2.546044
C	-0.344391	-5.190591	-1.426813
C	-0.866475	-4.274731	-0.519995
C	-2.953690	-2.529809	0.915197
C	-3.361213	-2.818141	2.219354
C	-4.606282	-3.400757	2.458993
C	-5.451650	-3.710481	1.397777
C	-5.046090	-3.440402	0.090931
C	-3.809929	-2.851331	-0.146406
C	-0.337574	-1.991148	2.025827
C	0.647193	-2.980532	2.088044
C	1.378770	-3.180687	3.259073
C	1.126171	-2.404360	4.385910
C	0.148406	-1.410968	4.332862
C	-0.567384	-1.198887	3.159208
C	-4.040505	0.724334	2.782532
O	0.338567	0.979061	-3.498120
C	3.309191	-0.749569	-2.763177
C	4.133092	-0.182388	-3.917851
Si	3.439591	0.202255	-1.166100
O	5.197580	0.284642	-0.919208
C	2.941852	2.004385	-1.264872
C	3.632544	2.957752	-0.287543
C	2.785995	-0.761466	0.289485
C	2.936610	-0.095139	1.654548
O	6.667701	-1.699978	-0.871517
O	6.669099	1.541999	0.725912

C	6.476406	1.823803	2.129353
H	5.641340	0.877107	-0.229437
C	6.928380	-2.031297	0.517291
H	5.764290	-0.596666	-0.952633
H	0.054344	0.372512	-0.912034
H	3.098138	2.344743	-2.298842
H	1.850520	2.012565	-1.107753
H	3.240215	3.977234	-0.380711
H	4.714240	3.015469	-0.468952
H	3.492637	2.649610	0.757402
H	3.981584	0.167349	1.875778
H	2.341120	0.825927	1.715096
H	2.589786	-0.755586	2.459651
H	1.722139	-0.957438	0.085230
H	3.281469	-1.747021	0.273124
H	2.242379	-0.762289	-3.033432
H	3.586114	-1.795165	-2.555899
H	3.840756	0.849381	-4.151626
H	5.206463	-0.175498	-3.688602
H	3.997871	-0.770922	-4.832080
H	-0.578203	0.788076	1.230300
H	0.843345	3.038942	0.609316
H	-0.022609	4.609656	0.616870
H	-0.517109	3.292156	1.744046
H	-3.306220	1.164227	3.470518
H	-5.016870	1.170691	3.015004
H	-4.106556	-0.351056	2.992664
H	-2.155360	5.582025	-0.780597
H	-4.179083	6.775156	0.034458
H	-5.915192	5.527305	1.307975
H	-5.644855	3.121413	1.745424
H	-3.340459	2.416411	-2.545110
H	-2.029963	1.708595	-3.560951
H	-3.744966	1.344365	-3.923212
H	-5.297700	-0.760834	-3.351068
H	-7.652627	-0.971069	-2.586267
H	-8.247076	-0.367838	-0.245324
H	-6.516318	0.464317	1.297043
H	-3.503911	-2.656108	-1.174679
H	-5.696028	-3.681933	-0.747422
H	-6.420803	-4.166788	1.585827
H	-4.908850	-3.617868	3.481073
H	-2.709509	-2.595831	3.062696
H	0.145258	-1.391339	-1.994964
H	1.070080	-3.020375	-3.627489
H	0.759458	-5.459119	-3.259872
H	-0.491597	-6.255589	-1.262674
H	-1.421247	-4.637529	0.344960
H	-1.311514	-0.404350	3.118647
H	-0.053208	-0.793825	5.205454
H	1.691991	-2.566400	5.300474
H	2.147436	-3.950040	3.285030
H	0.864578	-3.597865	1.218999
H	0.115996	0.852584	-2.553278

H	0.609111	1.900646	-3.574172
H	6.887823	-3.122746	0.618722
C	8.308334	-1.476808	0.799869
H	6.126224	-1.589416	1.121589
H	8.798503	-1.986871	1.633736
C	9.009466	-1.696401	-0.538089
H	8.243192	-0.404622	1.034259
H	9.890529	-1.062819	-0.675019
H	9.323155	-2.742629	-0.637854
C	7.916676	-1.371742	-1.539640
H	7.965040	-1.950526	-2.466011
H	7.889994	-0.300639	-1.790432
C	7.592927	2.784790	2.487213
H	5.484410	2.281631	2.260647
H	6.507873	0.873326	2.674929
H	7.342811	3.411560	3.347445
H	8.512361	2.230838	2.719216
C	7.759804	3.566300	1.187379
H	8.715853	4.091477	1.112271
C	7.621968	2.466600	0.155106
H	6.951394	4.301070	1.075272
H	7.236785	2.795249	-0.817516
H	8.571170	1.931775	-0.000985

- Compound: **C_2**

Symbol	X	Y	Z
C	-5.576430	-2.636713	-1.758271
C	-4.346842	-1.959181	-1.717451
C	-3.424486	-2.283492	-2.714692
C	-3.678589	-3.245213	-3.695793
C	-4.910354	-3.890089	-3.711134
C	-5.862361	-3.583000	-2.738224
Si	-3.902628	-0.671458	-0.359287
Ir	-1.715985	0.109478	-0.936843
P	-1.734296	1.811429	0.584537
C	-0.374674	3.062600	0.438500
C	0.150312	3.748210	1.539797
C	1.145305	4.708838	1.365264
C	1.610560	5.013467	0.087279
C	1.073677	4.355329	-1.018363
C	0.088546	3.385463	-0.842470
S	-1.796413	-1.489078	-2.727813
C	-1.925840	-0.521390	-4.265518
C	-3.687999	-1.730659	1.232399
C	-2.408100	-2.105762	1.653037
C	-2.181107	-2.844828	2.813353
C	-3.265350	-3.242688	3.590699
C	-4.556138	-2.887713	3.202671
C	-4.756851	-2.142229	2.042010
S	-0.951309	-1.618013	0.688575
C	-0.670148	-3.210724	-0.164457
C	-5.504508	0.308969	-0.093470
C	-3.157894	2.984385	0.551690
C	-3.448777	3.793311	1.657852
C	-4.475179	4.730877	1.596887
C	-5.208920	4.890131	0.421729
C	-4.908343	4.108805	-0.690350
C	-3.890811	3.159414	-0.623422
C	-1.642607	1.234869	2.324505
C	-0.408766	0.866110	2.876284
C	-0.344307	0.294345	4.144461
C	-1.511205	0.068876	4.871339
C	-2.744519	0.408368	4.320589
C	-2.810181	0.980782	3.053803
O	1.775554	0.820360	0.108325
Si	3.621873	-0.170755	-0.491657
C	4.469388	1.049209	0.673136
C	4.723221	2.420332	0.041768
C	3.300507	0.338744	-2.276784
C	2.572593	-0.749494	-3.069436
C	2.785934	-1.715052	0.187370
C	2.756896	-1.805372	1.711893
O	5.217491	-1.125076	-0.988378
O	6.024175	-2.569372	1.072228
O	7.104768	0.606598	-1.613443

H	-1.162565	-3.091694	3.114856
H	-3.100910	-3.813849	4.501643
H	-5.407831	-3.186904	3.810356
H	-5.775981	-1.868877	1.765832
H	-6.332120	-2.420452	-1.002182
H	-6.825585	-4.088744	-2.742013
H	-5.123691	-4.634262	-4.474787
H	-2.922104	-3.489596	-4.440703
H	-5.755124	0.875015	-0.999603
H	-6.354245	-0.349165	0.132863
H	-5.409134	1.030161	0.729585
H	-1.010864	0.072889	-4.336261
H	-1.995453	-1.204587	-5.115958
H	-2.798913	0.133686	-4.215018
H	0.116176	-3.064737	-0.911118
H	-0.342949	-3.945694	0.576810
H	-1.588476	-3.553443	-0.652092
H	-3.784601	1.228685	2.634531
H	-3.663082	0.215089	4.870289
H	-1.460357	-0.381327	5.860208
H	0.622288	0.018142	4.560901
H	0.510684	1.011728	2.308789
H	-0.202408	3.527512	2.545549
H	1.554993	5.221558	2.232466
H	2.389661	5.760435	-0.046435
H	1.427206	4.589088	-2.020233
H	-0.308665	2.849164	-1.704019
H	-3.672151	2.534469	-1.487254
H	-5.473072	4.228370	-1.612307
H	-6.010476	5.623640	0.374559
H	-4.698490	5.341763	2.468553
H	-2.880008	3.691305	2.580679
H	-2.226477	1.203343	-1.980925
H	-0.133029	0.421695	-1.539680
H	4.270787	0.556376	-2.747706
H	2.727218	1.275530	-2.304354
H	2.457687	-0.470755	-4.123686
H	3.116151	-1.702935	-3.038536
H	1.563758	-0.926349	-2.669852
H	3.268723	-2.602645	-0.246201
H	1.756344	-1.712289	-0.197824
H	2.226151	-2.705857	2.049719
H	3.772580	-1.843415	2.129550
H	2.242472	-0.941369	2.150871
H	3.855815	1.153776	1.579159
H	5.424406	0.613106	1.001709
H	5.213474	3.103342	0.746091
H	5.369184	2.336326	-0.842669
H	3.794090	2.909446	-0.282590
H	5.950800	-0.539634	-1.322816
H	5.581916	-1.703187	-0.262370
C	7.459268	1.369423	-2.782270
C	6.701446	-2.022972	2.219280
H	0.969074	0.590483	-0.429115

H	1.789384	1.789303	0.113134
C	6.592750	-3.091328	3.291194
H	7.748649	-1.825220	1.948394
H	6.220295	-1.073432	2.487002
H	7.404297	-3.031630	4.021878
H	5.639752	-3.001130	3.829095
C	6.612552	-4.369471	2.458170
H	6.211815	-5.239942	2.985221
C	5.761441	-3.971891	1.267768
H	7.636157	-4.602100	2.138032
H	6.007309	-4.499708	0.340723
H	4.688399	-4.102014	1.477585
C	8.479012	2.381294	-2.294543
H	7.885918	0.687713	-3.531133
H	6.546465	1.817952	-3.191572
H	9.145692	2.720228	-3.092462
H	7.974231	3.260215	-1.871493
C	9.190473	1.599518	-1.194248
H	9.733053	2.233968	-0.487781
C	8.030423	0.870183	-0.544149
H	9.899212	0.883833	-1.629888
H	8.301567	-0.085795	-0.080284
H	7.530305	1.501021	0.208224

- Compound: **TS2**

Symbol	X	Y	Z
C	0.508967	1.870482	-2.211502
C	-0.021278	2.508281	-1.087049
C	0.514320	3.740803	-0.691753
C	1.564646	4.315251	-1.401271
C	2.093470	3.665654	-2.516130
C	1.561973	2.444410	-2.921333
P	-1.406814	1.768050	-0.115881
C	-2.834577	2.824925	-0.568078
C	-2.863176	3.476363	-1.805663
C	-3.983670	4.210240	-2.189812
C	-5.084070	4.303953	-1.340964
C	-5.065518	3.652018	-0.108849
C	-3.951094	2.911286	0.272949
Ir	-1.612351	-0.509428	-0.446498
S	-1.583430	-2.818247	-1.132297
C	-1.290886	-2.791894	-2.932940
C	-3.289605	-3.415349	-1.111386
C	-4.326418	-2.505406	-0.901493
C	-5.627311	-3.029951	-0.876809
C	-5.868994	-4.389889	-1.053975
C	-4.805774	-5.269858	-1.259105
C	-3.502154	-4.784172	-1.282562
Si	-3.951228	-0.644965	-0.623451
C	-5.001584	0.255268	-1.902278
C	-4.609005	-0.271334	1.130928
C	-3.734742	-0.404474	2.218358
C	-4.101344	-0.064260	3.519675
C	-5.387466	0.416226	3.760265
C	-6.286849	0.543583	2.703262
C	-5.898014	0.201592	1.408496
S	-2.041878	-1.002602	1.964625
C	-2.312498	-2.786482	2.248879
C	-0.954978	2.276386	1.596391
C	0.043142	1.532890	2.238407
C	0.487480	1.884525	3.508171
C	-0.068081	2.983173	4.161578
C	-1.055230	3.734936	3.529688
C	-1.491312	3.389853	2.251463
O	1.444295	-2.075930	0.565993
Si	3.190580	-1.690437	0.089413
C	3.307228	-0.168026	1.197325
C	3.101255	1.150848	0.454827
C	3.973743	-3.294321	0.700161
C	4.375637	-4.254210	-0.419681
O	5.400195	-1.192791	-0.469782
C	2.761201	-1.507881	-1.749144
C	3.780501	-1.129862	-2.814647
O	7.013027	-1.654852	1.696503
O	6.124964	1.427617	-1.200904

C	5.727018	2.203163	-2.341656
H	5.619191	-0.300157	-0.806571
C	7.933885	-0.556741	1.798564
H	5.970275	-1.338492	0.316125
H	0.190572	-0.474571	-0.335469
H	2.293149	-2.473624	-2.008571
H	1.927355	-0.787488	-1.769831
H	3.316893	-1.112733	-3.809299
H	4.623178	-1.828040	-2.846516
H	4.193364	-0.128373	-2.637575
H	3.854615	1.285420	-0.333470
H	2.116936	1.199613	-0.030889
H	3.166499	2.016329	1.129289
H	2.556506	-0.281662	1.993512
H	4.286254	-0.175704	1.697466
H	3.264467	-3.782258	1.383476
H	4.847354	-3.037122	1.316011
H	3.509026	-4.561256	-1.020633
H	5.096615	-3.786555	-1.101594
H	4.835022	-5.169025	-0.025430
H	-1.523728	-0.132963	-1.996377
H	-0.303986	-2.346679	-3.089326
H	-1.307606	-3.818536	-3.308536
H	-2.061141	-2.183015	-3.414220
H	-4.613449	0.017046	-2.901363
H	-6.050643	-0.067343	-1.866765
H	-4.972028	1.343160	-1.774216
H	-2.661431	-5.461203	-1.428420
H	-4.990319	-6.333103	-1.391375
H	-6.888162	-4.768893	-1.025781
H	-6.473040	-2.361831	-0.707907
H	-3.101389	-3.171876	1.595176
H	-1.366123	-3.295686	2.044079
H	-2.587247	-2.929748	3.297295
H	-3.383307	-0.161025	4.333360
H	-5.680958	0.696932	4.768878
H	-7.289952	0.922650	2.886870
H	-6.608288	0.334833	0.591394
H	-3.960816	2.394873	1.232456
H	-5.923123	3.712331	0.558049
H	-5.956002	4.880946	-1.639497
H	-3.992903	4.712138	-3.154357
H	-2.008460	3.414058	-2.477657
H	0.481083	0.669142	1.738473
H	1.265990	1.295797	3.988994
H	0.271388	3.255131	5.158195
H	-1.486025	4.600625	4.027188
H	-2.245597	4.005549	1.766976
H	0.106583	0.909450	-2.524772
H	1.969935	1.929269	-3.789167
H	2.916874	4.114097	-3.069239
H	1.974376	5.269827	-1.078772
H	0.119064	4.256369	0.182603
H	0.623838	-1.246448	0.099345

H	1.141569	-2.963681	0.328044
C	9.279190	-1.187098	2.099146
H	7.956220	-0.018467	0.834593
H	7.566840	0.126428	2.572595
H	10.117887	-0.547597	1.808741
H	9.367359	-1.406800	3.170943
C	9.188748	-2.479229	1.293240
H	9.893868	-3.249772	1.618302
C	7.742265	-2.884762	1.518080
H	9.369474	-2.275817	0.229762
H	7.300968	-3.431212	0.673947
H	7.622366	-3.490344	2.427492
C	6.127764	3.629342	-2.017321
H	6.214615	1.784942	-3.228858
H	4.632632	2.121327	-2.468918
H	5.540460	4.367718	-2.572387
C	5.891371	3.670783	-0.510560
H	7.188883	3.792560	-2.245563
H	6.424054	4.480629	-0.003525
H	4.818661	3.778826	-0.297186
C	6.384166	2.298083	-0.086282
H	5.869836	1.896095	0.797858
H	7.466999	2.302373	0.110221

- Compound: **C_3**

Symbol	X	Y	Z
C	-3.480554	3.085748	0.019421
C	-2.313925	2.787647	-0.695172
C	-2.076132	3.431800	-1.912339
C	-3.006730	4.337442	-2.419865
C	-4.175561	4.611135	-1.714568
C	-4.406696	3.990193	-0.487567
P	-1.138433	1.581687	0.033308
Ir	-1.782396	-0.663878	0.240254
Si	-3.710514	-0.522729	-1.111210
C	-4.260735	-2.328157	-1.389299
C	-3.600896	-3.384319	-0.760494
C	-4.027517	-4.709960	-0.862215
C	-5.138881	-5.002437	-1.644888
C	-5.815493	-3.973166	-2.299612
C	-5.383163	-2.657271	-2.165175
S	-2.142924	-3.060578	0.250831
C	-0.839904	-3.747895	-0.826040
C	-0.656431	2.436870	1.588892
C	-0.617813	3.837808	1.625359
C	-0.128675	4.501254	2.745738
C	0.330680	3.775516	3.844042
C	0.288527	2.384336	3.820753
C	-0.207370	1.720656	2.700840
C	0.371595	1.724345	-1.004684
C	1.531876	2.344809	-0.532956
C	2.670287	2.411849	-1.335806
C	2.655596	1.872798	-2.617557
C	1.496836	1.266165	-3.102697
C	0.367425	1.181858	-2.297441
S	-3.345952	-0.242112	2.150815
C	-4.897864	0.284579	1.383264
C	-5.867789	0.819452	2.232389
C	-7.059654	1.292279	1.690865
C	-7.262319	1.239497	0.312497
C	-6.277071	0.711289	-0.517689
C	-5.069975	0.217956	-0.002535
C	-3.877905	-1.832378	2.871216
C	-3.755443	0.330455	-2.785293
O	1.659514	-2.905924	1.194799
Si	2.911898	-1.878094	0.690991
C	4.568304	-2.708058	1.001043
C	4.873146	-2.951260	2.478848
C	2.777365	-0.269230	1.662087
C	3.988479	0.649454	1.514418
C	2.560213	-1.600695	-1.122819
C	2.888106	-2.789413	-2.023389
O	5.666390	-0.400261	-1.558631
O	8.012044	-1.580275	-0.503746
O	5.906215	2.429797	-1.338146

H	5.335988	-2.067410	0.536540
H	1.721715	-3.258830	2.088066
H	-5.688273	0.879140	3.304997
H	-7.820633	1.713473	2.343092
H	-8.186224	1.621109	-0.116056
H	-6.448222	0.699563	-1.594416
H	-5.936492	-1.869300	-2.676504
H	-6.686857	-4.198317	-2.910033
H	-5.479307	-6.030615	-1.738202
H	-3.500126	-5.504756	-0.336805
H	-2.919333	-0.012849	-3.407036
H	-4.683005	0.091865	-3.321055
H	-3.691956	1.420554	-2.688955
H	0.113167	-3.544562	-0.324936
H	-0.997709	-4.826365	-0.909853
H	-0.882612	-3.266276	-1.806428
H	-2.994021	-2.304127	3.308685
H	-4.615314	-1.630019	3.651813
H	-4.311213	-2.477688	2.101071
H	-1.164996	3.234799	-2.473005
H	-2.812055	4.830688	-3.369062
H	-4.900831	5.316144	-2.113467
H	-5.309327	4.208676	0.078594
H	-3.667627	2.625567	0.989100
H	-0.957144	4.417402	0.768146
H	-0.101920	5.588095	2.757872
H	0.716692	4.294936	4.717739
H	0.639015	1.809883	4.674951
H	-0.234441	0.633773	2.705570
H	-0.523309	0.682711	-2.678091
H	1.478855	0.839252	-4.102749
H	3.552115	1.909282	-3.233613
H	3.583025	2.866436	-0.952496
H	1.568352	2.765337	0.470744
H	-0.978656	-0.884272	-1.118095
H	-0.510562	-0.995623	1.580513
H	3.141800	-0.722639	-1.439444
H	1.498246	-1.317464	-1.217141
H	2.575140	-2.614298	-3.060943
H	3.969367	-2.977728	-2.039212
H	2.397440	-3.712483	-1.683532
H	4.588333	-3.656486	0.441662
H	1.877189	0.253647	1.299476
H	2.584481	-0.495502	2.723537
H	3.820779	1.627236	1.988740
H	4.887794	0.213707	1.972005
H	4.228212	0.827831	0.456107
H	-0.086983	-1.110910	0.874761
H	5.845553	-3.439730	2.630499
H	4.893470	-2.012267	3.048821
H	4.120896	-3.599209	2.948892
H	5.733997	0.569217	-1.599777
H	6.522652	-0.705511	-1.208542
C	6.408898	2.738085	-0.031387

C	7.967826	-2.957085	-0.912334
C	7.258454	3.983835	-0.202428
H	5.556649	2.927307	0.642647
H	6.956520	1.864062	0.345285
H	7.336302	4.569363	0.718518
H	8.272415	3.717809	-0.528400
C	6.511538	4.699262	-1.323759
H	7.108769	5.456141	-1.840749
C	6.133777	3.535523	-2.222360
H	5.607673	5.183906	-0.928852
H	5.221210	3.707482	-2.807235
H	6.948470	3.278896	-2.915960
C	8.032921	-3.781434	0.365040
H	7.048039	-3.121764	-1.490669
H	8.828738	-3.149869	-1.567843
H	8.529361	-4.744738	0.214431
C	8.785765	-2.849306	1.310423
H	7.021844	-3.976347	0.747468
H	8.622819	-3.075674	2.369152
H	9.864808	-2.890363	1.113211
C	8.230287	-1.495835	0.907964
H	8.910398	-0.658857	1.099764
H	7.270721	-1.287262	1.413639

- Compound: **C_4**

Symbol	X	Y	Z
C	8.027633	-1.285137	0.704150
O	7.834651	-1.409817	-0.707881
C	7.866360	-2.795417	-1.087362
C	7.976242	-3.588357	0.207072
C	8.662681	-2.592863	1.138616
O	5.457272	-0.331715	-1.797716
O	5.799311	2.481130	-1.455174
C	6.389817	2.631851	-0.157282
C	7.311239	3.833766	-0.261472
C	6.564522	4.693947	-1.275709
C	6.069536	3.641862	-2.251562
C	2.585344	2.636284	-1.287129
C	1.469182	2.504594	-0.462385
C	0.339202	1.812432	-0.907845
C	0.341752	1.269287	-2.199375
C	1.447005	1.421007	-3.028876
C	2.577813	2.094090	-2.567936
P	-1.129823	1.560552	0.169842
C	-0.592936	2.191198	1.804352
C	-0.478986	3.563578	2.061484
C	0.020879	4.009734	3.280521
C	0.414894	3.091249	4.253728
C	0.300124	1.725551	4.008673
C	-0.206386	1.279713	2.790185
C	-2.334443	2.826910	-0.388346
C	-3.346660	3.234112	0.490359
C	-4.301974	4.157846	0.083741
C	-4.266182	4.677320	-1.209951
C	-3.262690	4.279153	-2.088367
C	-2.295798	3.362082	-1.678259
Ir	-1.776996	-0.682714	0.268707
S	-3.579388	-0.272888	1.973994
C	-4.368753	-1.830730	2.509449
Si	-3.506354	-0.575300	-1.237500
C	-3.220238	0.016764	-2.992989
S	-2.027331	-3.076084	0.242260
C	-0.847059	-3.708386	-0.997252
C	-4.199237	-2.347678	-1.315454
C	-3.570343	-3.392371	-0.636070
C	-4.087572	-4.687721	-0.605062
C	-5.269298	-4.957772	-1.288431
C	-5.920857	-3.936944	-1.980686
C	-5.392691	-2.648595	-1.987033
C	-4.876280	0.421150	-0.390312
C	-4.925710	0.450526	1.008821
C	-5.957895	1.080799	1.701750
C	-6.977165	1.702250	0.984352
C	-6.941603	1.709136	-0.408902
C	-5.901046	1.075441	-1.085118
O	1.532085	-2.889470	1.119959

Si	2.782490	-1.882092	0.571565
C	2.415067	-1.621332	-1.242009
C	2.770926	-2.801322	-2.143785
C	4.444065	-2.715636	0.854506
C	4.785220	-2.936626	2.327549
C	2.685326	-0.270431	1.533666
C	3.887517	0.653626	1.358659
H	5.198601	-2.077166	0.363795
H	1.633603	-3.257442	2.003340
H	-5.957659	1.100372	2.790732
H	-7.787135	2.197004	1.514802
H	-7.725563	2.213171	-0.969589
H	-5.886915	1.095500	-2.175389
H	-5.925645	-1.860722	-2.521014
H	-6.847505	-4.146057	-2.509926
H	-5.684590	-5.962320	-1.274201
H	-3.574925	-5.473750	-0.052515
H	-2.417714	-0.571278	-3.455668
H	-4.128017	-0.134420	-3.592244
H	-2.957076	1.080295	-3.039902
H	0.148625	-3.524868	-0.582244
H	-1.020884	-4.781055	-1.119755
H	-0.987377	-3.175702	-1.942493
H	-3.611947	-2.408921	3.046478
H	-5.194575	-1.590436	3.183791
H	-4.735952	-2.393066	1.645109
H	-1.504529	3.078154	-2.370081
H	-3.220835	4.689142	-3.094615
H	-5.016633	5.396705	-1.529008
H	-5.078487	4.468987	0.778905
H	-3.383685	2.842099	1.506519
H	-0.772384	4.287864	1.302576
H	0.106876	5.076975	3.469557
H	0.808240	3.442099	5.204785
H	0.603034	1.004320	4.764084
H	-0.289600	0.207582	2.610771
H	-0.516912	0.700654	-2.554441
H	1.435906	0.986841	-4.025949
H	3.460544	2.177019	-3.199073
H	3.478916	3.137995	-0.917904
H	1.504362	2.920348	0.542765
H	-0.706468	-0.896221	-0.888754
H	2.971840	-0.728243	-1.562155
H	1.345839	-1.366231	-1.326906
H	2.438791	-2.641101	-3.177671
H	3.857447	-2.954263	-2.173757
H	2.317762	-3.740855	-1.796227
H	4.457671	-3.671011	0.307234
H	1.770747	0.244796	1.197420
H	2.522637	-0.498744	2.599509
H	3.733157	1.621985	1.857352
H	4.803368	0.212393	1.777688
H	4.088857	0.854160	0.296293
H	5.763136	-3.418744	2.464968

H	4.813818	-1.990164	2.884999
H	4.047324	-3.580993	2.825034
H	5.509562	0.639309	-1.814985
H	6.315218	-0.625229	-1.441679
H	5.587796	2.805142	0.579394
H	6.899325	1.694740	0.105169
H	7.469765	4.326414	0.702483
H	8.289408	3.536325	-0.661288
H	7.187873	5.454942	-1.754469
H	5.714409	5.197654	-0.794549
H	5.149180	3.925612	-2.777765
H	6.836403	3.394453	-3.001083
H	6.956680	-3.023065	-1.660655
H	8.736131	-2.952997	-1.740220
H	8.533492	-4.521050	0.078186
H	6.977308	-3.839376	0.588289
H	8.504758	-2.808043	2.200460
H	9.743766	-2.570477	0.950063
H	8.650413	-0.402365	0.887227
H	7.050295	-1.130190	1.195605
H	5.386207	-3.310683	6.050300
H	5.891752	-3.216766	5.513920
